# Fertility among Norwegian Women and Men with Mental Disorders

**DOI:** 10.1007/s10680-025-09739-5

**Published:** 2025-07-16

**Authors:** Øystein Kravdal, Martin Flatø, Fartein A. Torvik

**Affiliations:** 1 Centre for Fertility and Health, Norwegian Institute of Public Health, Oslo, Norway; 2 Department of Economics, University of Oslo, Oslo, Norway

**Keywords:** Fertility, Men, Mental health, Register data, Women

## Abstract

We analysed the associations between mental disorders and women’s and men’s first-, second- and third-birth rates in 2010–2018, using register data for the full Norwegian population. Having had at least one consultation for depression in primary or specialised health care in the calendar year before the preceding year was associated with lower birth rates. According to a simulation, the birth-rate reduction corresponds to a reduction in completed fertility from 1.60 births per woman among those without any of the mental disorders under study, to 1.34 among those with depression throughout the reproductive period. The corresponding numbers for men were 1.41 and 0.90. The associations between anxiety and fertility were of similar magnitude, while fertility was even lower among individuals with bipolar disorder, eating disorder or personality disorder. The simulated completed fertility was lowest among women and men with schizophrenia (0.36 and 0.16, respectively). However, to the extent that individuals with these mental disorders enter parenthood, many of the estimates suggested that they do so at a relatively early age. The associations between mental disorders and fertility became weaker when partnership, education and income were controlled for, and when characteristics shared by siblings were taken into account in family fixed-effects models.

**Supplementary Information:**

The online version contains supplementary material available at 10.1007/s10680-025-09739-5.

## Introduction

The prevalence of depression, anxiety and other mental disorders among adolescents and young adults appears to have increased over the last few decades in Norway and other high-income countries (Bor et al., 2014; Galmiche et al., 2019; Krokstad et al., 2022; Centers for Disease Control and Prevention, 2023). At the same time, fertility has declined in many countries, including Norway where the total fertility rate fell from 1.98 births per woman in 2009 to 1.40–1.44 in 2022–2024—to a large extent because of reduced first-birth rates among the relatively young (Hart & Kravdal, 2020; Statistics Norway, 2025a).

An important question from a demographic perspective is whether a rise in mental disorders, which we may also see in coming years, may have implications for fertility. However, this issue has received very little attention in demographic journals. The quite few studies of the importance of health as a fertility determinant in high-income settings have largely dealt with general health (Alderotti & Trappolini, 2021; Barclay & Kolk, 2020; Dommermuth et al., 2011; Fiori et al., 2017; Gray et al., 2013; Syse et al., 2022). In contrast, medically oriented journals include several papers on how fecundity or fertility appears to be influenced by specific somatic (Ban et al., 2015; Dumanski & Ahmed, 2019; Ferraro et al., 2017; Gade et al., 2014; Wiebe et al., 2014) or mental diseases. Most of the investigations have shown relatively low fertility among individuals with mental disorders, although the associations are stronger for some of these disorders than others (Bundy et al., 2011; Laursen & Munk-Olsen, 2010; Power et al., 2013), and there is evidence of high fertility at low age (Laursen & Munk-Olsen, 2010; Jokela et al., 2014; Evensen & Lyngstad, 2020).

In this study, we use data from Norwegian registers covering the period 2010–2018 to analyse how individuals’ birth probabilities (or rates) are associated with previous mental disorders, as indicated by healthcare consultations two years earlier. We consider depression and anxiety, which are the most common mental disorders. Additionally, we consider schizophrenia, bipolar disorder, eating disorder (only among women) and personality disorder, which are also relatively common. These disorders cover in total the main types of mental health problems (Kotov et al., 2017) except externalising disorders. We have not analysed externalising disorders such as alcohol use disorder and ADHD, as they are hard to identify in our register data (see Newcorn et al. [2007] for a discussion of ADHD among adults).

Using these data, we are able to address several limitations in the existing literature. First, previous studies have not always focused on associations between indicators of mental health in a certain period and *subsequent* fertility, but rather the associations between corresponding cumulative measures, which leaves more doubt about the direction of causality (Power et al., 2013). Second, earlier studies of health effects on fertility that have been based on healthcare registers have only taken specialised healthcare into account. In the Norwegian healthcare system, which is public with a quite small private supplement (Saunes et al., 2020), specialised treatment is contingent on referral from a general practitioner (GP), who typically takes responsibility for the least severe cases. Because we include GP consultations, our results are plausibly more generalisable to everyone with the mental disorder, many of whom never seek professional treatment. We check how this broader disease definition based on both primary and specialist healthcare influences the results.

Third, we consider both sexes. This has not always been done in earlier studies, and those that have addressed the sex differences have provided conflicting evidence (Kailaheimo-Lönnqvist et al., 2024). Fourth, we separate between first, second and later births, as in only one earlier study (Laursen & Munk-Olsen, 2010), and we distinguish between first births in different age groups. Fifth, we check whether the associations are partly driven by partnership status, education and income, which may not only be selection factors, but also mediators. The role of these factors has attracted little attention earlier. Sixth, we show the implications of including family fixed effects, to control for all constant characteristics shared by siblings. This has not been done in previous investigations of the associations between mental health and fertility, except in a recent paper on lifetime childlessness based on data from specialised healthcare (Liu et al., 2024).

Finally, we take a novel step by predicting—by means of stochastic simulation—how the estimated age- and parity-specific regression coefficients for the different disorders add up to differences in average completed fertility, proportion childless at various ages, and average age at first birth. These differences may be more easily interpretable for most readers than the differences in birth rates. As part of this step, we also calculate how much the disorders in total contribute to completed fertility at the national level. The fact that we capture a larger proportion of individuals with these disorders by using information about primary and not only specialised healthcare makes these calculations more reasonable.

The study contributes to the understanding of how mental health is associated with fertility, and how changes in mental health therefore may have influenced past fertility trends and may shape the future fertility development. However, knowledge about fertility among individuals with mental disorders is also valuable beyond this perspective, for several reasons. For example, if old people with serious mental (or somatic) chronic diseases have no or few children, they may need particularly much support from health institutions (Deindl & Brandt, 2017). Furthermore, although some individuals with large health problems may have decided against childbearing because they think this is in their best interest under the circumstances, not having children may nevertheless be felt as a loss that adds to their burden. Additionally, parents’ health may affect the children’s health through social processes or genetic transmission (Gjerde et al., 2021), and knowledge about how health affects fertility therefore contributes to our insight into how the distribution of health problems in the population changes over generations and time.

## Background

### Studies of Associations Between Mental Disorders and Fertility

Studies have shown low fertility among individuals with schizophrenia, and a less markedly reduced fertility associated with bipolar disorder or eating disorder (Bundy et al., 2011; Laursen & Munk-Olsen, 2010; Power et al., 2013). Reduced fertility has also been observed among individuals with depression. Some investigations have shown this most clearly or only among men (Golovina et al., 2023; Power et al., 2013), while other researchers have reported that use of antidepressants is most strongly associated with childlessness among women (Kailaheimo-Lönnqvist et al., 2024). Some smaller studies have shown no or (for young women) a positive association with fertility (Grundström et al., 2023; Jonsson et al., 2011; Nilsen et al., 2012). Studies of individuals with internalising disorders, which include depression and anxiety, indicate reduced fertility primarily among men, and additionally suggest that the reduction occurs especially among the relatively old, while birth rates at lower ages may be higher than among those without such disorders (Laursen & Munk-Olsen, 2010; Jokela et al., 2014; Evensen & Lyngstad, 2020). The study of individuals with depression by Golovina et al. (2023) also indicates low age at first birth (among those becoming parents), and even stronger evidence of early childbearing has appeared in analyses of externalising behaviour (Jokela et al., 2014; Østergaard et al., 2017; Evensen & Lyngstad, 2020). Studies of larger groups of mental disorders have pointed in the same direction (Selling et al., 2009; Vigod et al., 2014).

### Possible Reasons for Effects of Mental Health on Fertility

#### Four Main Proximate Determinants

According to the framework by Easterlin and Crimmins (1985), the following may be considered as four main determinants of a person’s probability of having a child, or another child: whether they are in a romantic relationship, fecundity (broadly defined), childbearing desires, and whether contraception is used adequately if a child is not wanted. The childbearing desires may in turn be reckoned as determined by purchasing power, direct and opportunity costs of childrearing, and preferences (‘tastes’) for spending time and money on children rather than other sources of satisfaction. Mental health may affect fertility through all these four main channels.

#### Effects Involving Income and Education

The effects of mental health on partnership, fecundity, childbearing desires and contraception may partly operate through income and education: Mental disorders may lead to lower education and income (Evensen et al., 2017; Kessler et al., 2008; Nordmo et al., 2022), which in high-income countries may have implications especially for partnership and childbearing desires, and perhaps to some extent fecundity.

To start with partnership, high income is commonly thought to increase the probability of entering cohabitation or (to larger extent) marriage, and of remaining in the union, while women’s income may have a less positive impact, although this sex difference seems to be diminishing (Kalmijn, 2013; Kravdal, 1999; Lyngstad & Jalovaara, 2010; Sweeney, 2002). High income may also affect fecundity, broadly defined, by making assisted reproductive technology more available (Goisis et al., 2020).

With regard to the income effects on childbearing desires, two counteracting mechanisms are widely accepted as relevant (Butz & Ward, 1979). First, a higher purchasing power may strengthen the interest in having children, although this is less obvious if it also increases the spending on each child. Second, having higher wages means that more income is foregone (i.e. opportunity costs are higher) per time unit a parent stays home with a child without paid parental leave. Given women’s stronger involvement in childcare, one would expect to see a more negative or less positive fertility effect of women’s earnings than men’s earnings (but less clearly in settings where childcare can be purchased).

In support of these ideas, empirical studies have shown non-negative associations between a man’s income and fertility (Hart, 2015; Kolk, 2023; Kornstad & Rønsen, 2018; Trimarchi & van Bavel, 2020), without identifying whether this is a result of differences in partnership, childbearing desires or other factors. Some have also shown positive associations between women’s earnings (potential) and fertility, while others have shown the opposite (Kornstad & Rønsen, 2018; Trimarchi & van Bavel, 2020).

Education is closely linked to income, but may affect fertility (via the main determinants) also through other factors, such as having flexible work or being well-oriented about relevant issues. Existing evidence suggests that higher education level in total may have a positive impact on fertility among men in particular (Jalovaara et al., 2019; Kravdal & Rindfuss, 2008).

#### Other Types of Effects

In addition to influencing fertility through income and education, mental disorders may have other types of effects. For example, the lower probability of union formation and stability among individuals with mental disorders observed in some studies (MacCabe et al., 2009) might partly reflect that they are less attractive as partners because of behaviours that complicate everyday life, or because they need emotional and other support. Additionally, a less active social life may reduce their chance of finding partners.

Mental disorders may also influence fecundity, including sexual activity, through biological mechanisms. Studies have shown associations between weak sexual desires and depressive symptoms (Lourenco et al., 2010), and reduced semen quality among men under psychological stress (Bhongade et al., 2015). It has also been reported that women with eating disorders have reduced fecundity (Hecht et al., 2022).

Furthermore, mechanisms beyond the economic may contribute to the lower childbearing desires observed among individuals with mental disorders (Carlsson & Kim, 2024). For example, they may see childbearing as less emotionally rewarding (i.e. they have less strong ‘*tastes’* for it) in a wide sense of that concept: They may fear that they will be too tired to enjoy the parental role or that the child will inherit the disease, or they may be burdened by doubts about their ability to care for the child (Shover, 2005; Schmidt et al., 2016; Ferraro et al., 2017; Meaney, 2018; Harpe et al., 2022). There may also be concerns about whether the recommended medication for, for example, bipolar disorders or depression may affect a child negatively (Desaunay et al., 2023; Munk-Olsen et al., 2018; Suarez et al., 2022).

Finally, some mental disorders may reduce the probability of using adequate contraception, through psychological mechanisms (Hall et al., 2015). Accordingly, unintended pregnancies have been shown to be relatively common among individuals with depression, anxiety or anorexia (Hall et al., 2015; James-Hawkins et al., 2014; Micali et al., 2014) and elevated abortion rates have also been reported (Laursen & Munk-Olsen, 2010).

To summarise, a variety of causal pathways, some of them involving lower education and income, may—through partnership, fecundity, childbearing desires or contraceptive use—contribute to a smaller number of children among individuals with mental diseases. However, there are also counteracting mechanisms. The latter may be most important for women because they often are the main caretakers, which means that low education and income may have less adverse impact on partnership and childbearing desires for them. Less severe symptoms among women than men (Bundy et al., 2011; Laursen & Munk-Olsen, 2010) may also contribute to a less negative impact of mental disorders on partnership and childbearing desires for them.

### Possible Effects on Fertility Timing

A mental disorder may lead to postponement of fertility, or the opposite, in addition to affecting the number of children born. This ‘timing’ issue is particularly relevant in analyses of first births, because the vast majority want to become parents (Sturm et al., 2023), while decisions about higher-parity births to a large extent are about whether to have another child at all (‘quantum’). If the effect of a disorder on the first-birth rate is more negative at low age than at high age, it indicates that the disorder leads to later first birth, given that a first birth occurs. Conversely, the opposite pattern across age (an increasingly negative effect) indicates a tendency to enter parenthood relatively early.

To start with some possible effects through socioeconomic resources, low education and income among individuals with mental illness may not only reduce their probability of forming a union (most markedly among men); it may also affect the timing. Studies focusing specifically on this have shown early union entry among the less educated, perhaps to a large extent because of fewer years spent in school (Manning et al., 2014).

It may also be argued that individuals with relatively low income will be particularly interested in postponing parenthood. One reason is that they may be more likely than those with higher income to expect an increase in income and purchasing power (see Happel et al. [1984] for explanation of why postponement then would be rational). A related reason is that, given the magnitude of a future income rise, it would be more valuable to wait with the childbearing until it has taken place if the starting point is low (because of the diminishing marginal utility of purchasing power). A counter-argument is that, if there is a particularly large income rise, birth postponement will also increase the short-term opportunity costs of childbearing particularly much. This is relevant especially with respect to women’s income. Studies of income effects on fertility are usually not designed to separate timing and quantum effects, but the overall effects (without regard to age) that appear in analyses of first births at least provide some support for the idea that low income among men leads to later parenthood (Hart, 2015).

Furthermore, the fewer years spent in school among those ending up with a low education level is believed to influence their desires about first-birth timing (Kravdal, 1994). One argument is that childbearing costs may be particularly high if a man or woman has a child while still in education, because in such a situation they may not be able to reach their educational goals, which would have negative implications for their long-term income (see Happel et al. [1984] and Gustafsson [2001] for arguments about timing-dependent childbearing costs). In other words, those with few years in school may want relatively early childbearing. The lower education level may also itself have an impact on timing, net of income, for reasons not further discussed here.

To give some examples of possible timing effects not involving education or income, individuals with mental disorders may, as mentioned, face special non-economic burdens of childrearing, and they may want to synchronise their first birth with their expected health situation: Given that they want to become parents, they may want to postpone this transition if they expect an improvement. If they instead expect a worsening of their condition, it is in theory possible that they would have children sooner rather than later to take advantage of relatively healthy life years. It is also possible that a tendency to use contraception inadequately may not only increase the number of children, as mentioned earlier, but lead to relatively early childbearing.

Furthermore, one may observe age differences in the associations between mental disorders and fertility for the following reasons: First, the group with a mental health diagnosis consists of some individuals with severe symptoms and others who are less affected. If, at any age, the probability of entering parenthood within the next year declines with worsening symptoms, those who have a diagnosis and are still childless at, say, age 30 will include a larger proportion with severe symptoms than those who have a diagnosis and are childless at, say, 20. Thus, the estimated effect of having a mental health diagnosis becomes more negative as age increases. Stated differently, to the extent that they enter parenthood at all, individuals with such a diagnosis will seem to do it relatively early. Second, among individuals diagnosed already by age 20, the disease is particularly likely to have started early, which is linked to high severity (Kessler et al., 2001; Lahey et al., 1999). This mechanism contributes in the opposite direction.

To summarise, one may expect individuals with mental disorders to become parents relatively early because of, especially, fewer years in education and more unplanned pregnancies. As mentioned, such a link between mental disorders and early parenthood has indeed been shown in earlier statistical analyses. However, there are also arguments pointing in the opposite direction. In particular, to the extent that men with mental disorder have low income, relatively late fatherhood might be expected.

### Variations Across Parity

Although the possibility of parity-dependent effects has attracted little attention (Laursen & Munk-Olsen, 2010), such variation is indeed plausible, and may go in either direction. For example, a reduced probability of finding a partner among individuals with mental disorders has implications especially for the first-birth rates. However, these individuals may also be more likely to experience union dissolution (Breslau et al., 2011), which is relevant at higher parities. Furthermore, while having the first child typically is the most life-changing transition, it is possible that the burden of having another child—which may be particularly heavy for individuals with mental disorders—may be considered larger at parity two than at parity one. Finally, there is a selection similar to that relevant for timing: Among individuals with a mental disorder, the disorder may be less severe among those who already have a child. Thus, the estimated effect of the disorder may be weaker for second- and higher-order births.

### Variables Included in the Analysis and Selection Issues

It is not possible, given the available data, to analyse the importance of the potentially mediating factors in detail. For example, there is obviously not information about childbearing desires in register data. However, the data allow us to consider partnership as well as education level, school enrolment and income—the latter three operating through both partnership and other channels. A complicating factor is that partnership and socioeconomic characteristics may also be among the joint determinants of mental health and fertility (Silva et al., 2016). In other words, they may be both mediators and selection factors.

There are also other factors that ideally should be controlled for because they may be joint determinants of mental disorders and fertility. For example, personality is related to—and possibly influencing—mental health, and may additionally affect fertility, partly through social relations (Kotov et al., 2010). It is also possible that genetic liability for certain mental disorders may be linked to fertility, although this did not show up in a recent analysis of schizophrenia (Lawn et al., 2019). Furthermore, mental health problems may be partly a result of sub- or infecundity, which obviously influences later fertility, and there is an ongoing discussion about whether hormonal contraceptive use—which clearly may affect fertility—increases depression risks (Fruzzetti & Fidecicchi, 2020). Finally, it is possible that experience of parental divorce, the parents’ socioeconomic resources and health, and various characteristics of the place of residence in childhood may influence both mental health and fertility (Kinge et al., 2021; Silva et al., 2016). In this study, we control for genetic, sociodemographic, and other family background factors that are shared by maternal siblings.

## Data and Methods

### Data Sources

The key data sources were the Norwegian Population Register, the KUHR register, and the Norwegian Patient Register, from which data covering the period up to 1 January 2019 were extracted. (KUHR is the acronym for The Norwegian Control and Distribution of Health Reimbursement Database).

All persons who have ever lived in Norway after 1964 have been included in the Population Register and assigned a personal identification number (PIN) that is also used in other registers. The Population Register includes information about the person’s sex, country of birth, and dates of birth and (if occurred) death. From 2005, there is information on marital and cohabitation status as of 1st January for everyone. Additionally, the data allow construction of almost complete birth histories for women and men born in Norway after 1935, and they include annual information on municipality of residence as of 1st January, with a missing code for residence abroad.

The KUHR register includes information about consultations with GPs from 2006. Up to two (or in a few cases three) diagnoses or symptoms, in the ICPC-2 system, are reported for each consultation. Our construction of disorder indicators was based on only face-to-face GP consultations and the diagnoses (not symptoms).

The Norwegian Patient Register (NPR) includes data on use of specialised healthcare from 2008 or, for some types of specialised care, a later year. We took all consultations into account, and considered both main and secondary diagnoses.

Additionally, the analysis was based on annual data from Statistics Norway on income, school enrolment, and education level.

See Appendix 1 in the online supplement, for further details about the data and methods.

### Discrete-time Hazard Models

Discrete-time hazard models for first, second and third births (and in some parts of the analysis higher-order births) were estimated separately for women and men born in Norway between 1965 (who were 45 years in 2010) and 2001 (17 years in 2018). In the analysis of first births, a series of 3-month observations (January–March, April–June, July–September, or October–December) was constructed for each individual. The first quarterly observation started 1st January the year the individual turned 17 or 1st January 2010, whichever came last. (This means that for individuals who were older than 17 years in 2010, the series of observations may be described as left-truncated.) The reason for starting the analysis no earlier than 2010 is that the first year with information on specialist consultations is 2008, and that our mental disorder indicators, which are based on consultations, are lagged two years in most of the analysis (see below). The analysis was further restricted to individuals who had lived in Norway 1st January every year from 2008 to the year of the first quarterly observation, in order to make the disorder indicators as relevant as possible (see elaboration in Appendix 1 in the online supplement). The last observation was the last quarter of the year when the individual turned 45, the last quarter of 2018, the last quarter of the year when the individual emigrated for the first time after 2008, the quarter before the individual died, or the quarter when the first child was born, whichever came first. Each 3-month observation included independent variables (described below) and an outcome variable, which was whether a first birth took place within the 3 months. The calendar year including the quarter is referred to as t below. The logistic model

$${\text{log}}\left( {{\text{p}}_{{{\text{iq}}}} /\left( {{1} - {\text{p}}_{{{\text{iq}}}} } \right)} \right) \, = {{\mathrm{\upbeta}}}_{0} + {{\mathrm{\upbeta}}}_{{1}} {{X}}_{{{\text{it}}}}$$

was estimated from all 3-month observations for all individuals. p_iq_ is the probability that individual i had a first birth within quarter q in year t, β_0_ is a constant term, X_it_ is a vector of categorical variables further described below, and β_1_ is the corresponding coefficient vector. We report the exponentials of β_1_ in the tables and text below. They are the ratios of the odds of a first birth for a certain category of a variable relative to the odds of a first birth for the reference category of that variable, all other variables fixed. Note that, because the probabilities of having a child within a quarter are generally quite small, the odds (ratios) do not differ much from the corresponding probabilities (ratios). In accordance with common practice, we often refer to the odds of having a birth as the ‘birth rate’ (although strictly speaking, a rate or hazard is a probability per time unit).

The analysis of second or third births was similar, except that the first 3-month observation was no earlier than the quarter after the previous birth. In total, 587,718 women and 708,456 men contributed to the analysis of first births, and the corresponding numbers were 268,977 and 264,835 for the second births and 326,463 and 281,641 for the third births.

Parts of the analysis of first births were stratified by the age at the end of year t, using five age groups. Variation in fertility effects across age provides information about timing. For example, if a disorder increases first-birth rates at ages 17–26 and reduces first-birth rates at higher ages correspondingly, so that the probability of ever becoming a parent remains unchanged, it means that individuals with the disorder become parents earlier.

Because there are probably joint unobserved determinants of first, second and higher-order birth rates, one might consider it more reasonable to estimate one so-called multilevel-multiprocess model that includes all the parity transitions, such as in some other fertility studies published over the last couple of decades (Kravdal, 2001). However, this much more complex analysis did not give markedly different results (Appendix 2 in the online supplement).

### Variables

We included one dichotomous indicator for each disorder. It was 1 (0) if the disorder was (not) included as at least one of the diagnoses for either a GP consultation or a specialist consultation in year t-2 (i.e. the calendar year two years before the year of the quarter we observe the fertility outcome). This year was chosen to (i) avoid capturing a reverse-causality effect *of* pregnancy *on* mental health, and (ii) observe mental health as close as possible to the fertility outcome in order to increase its relevance. Note that individuals who did not have a consultation for the disorder in t-2 may have had such a consultation earlier, and they may no longer have symptoms or want to seek help for them in t-2. However, the vast majority of those without a consultation in t-2 do not have, and have not had, the mental disorder (although they may have others). The diagnosis codes for the various disorders are shown in Table 1. Alternative health indicators—partly involving longer lags—were used in some models (see below).

**Table 1:** Definitions of mental disorder indicators based on ICPC-2 codes in primary health care and ICD-10 codes in specialised health care

Dichotomous disorder indicators	ICD-10	ICPC-2
Depression	F32, F33	P76
Anxiety	F41	P74
Schizophrenia	F20	P72
Bipolar disorder	F31	P73
Eating disorder	F50	P86
Personality disorder	F60, F61	P80

We estimated different versions of the first-, second-, and third-birth models. In one version, we only included—in addition to the disorder indicators—the age at the end of year *t*, duration since last previous birth (when relevant), and t itself. In other versions, we included these three control variables plus one or more of the following categorical variables reflecting sociodemographic characteristics of the index person:

(i) Partnership status at the beginning of t-1 (married, widowed and not cohabiting, divorced and not cohabiting, separated and not cohabiting, never-married and not cohabiting, or cohabiting),
(ii) The highest education level attained as of 1st October in t-2 (primary or not completed higher secondary, higher secondary, lower tertiary, higher tertiary, or missing information),
(iii) Whether the individual was enrolled in school 1st October in t-2, and
(iv) The annual income in NOK in t-2 (0, 1–49,999, 50,000–99,999, 100,000–149,999, etc., up to 950,000–999,999, ≥ 1,000,000, or missing information).

As explained above, the index person’s partnership status, education and income may influence both their mental health and fertility, in which case it would be reasonable to control for these factors. However, they may also be causally between mental health and fertility, so that we are ‘taking away’ a mediating channel by controlling for them. This problem (which cannot be fully solved by using appropriate lags; see Appendix 1 in the online supplement) is the reason why we estimate both models including these variables and models not including them.

### Family Fixed Effects

Part of the analysis is based on family fixed-effects models, to control for constant (observed and unobserved) characteristics shared by maternal siblings. Such models have been estimated only in a few studies of fertility effects of physical diseases (Green et al., 2009; Penovich, 2000; Wiebe et al., 2014) and in the recent study of childlessness that included both mental and physical diseases registered in specialised health care (Liu et al., 2024). It is not obvious that family fixed effects can be included in a discrete-time hazard model, but it has been shown that they can be included in a Cox model (Ridder & Tunali, 1999). This is another (and continuous-time) type of hazard model, which we do not use as our main tool because it does not include a specified baseline hazard that makes it possible to do the simulation.

Fortunately, the estimates from Cox models without family fixed effects were almost identical to those from the discrete-time models (see below). Before adding the fixed effects, we restricted the dataset to individuals who had a same-sex maternal sibling under exposure for the same fertility transition within the relevant period.

In principle, estimates from a sibling model will be biased if fertility is affected by both own and a sibling’s mental health, but as discussed in Appendix 3 in the online supplement, there is little reason for concern about this.

### Simulations

In each simulation, we generated birth histories for 100,000 women. This was a sufficiently large dataset, because we got the same results with twice as many individuals. Starting at age 17, a 3-month birth probability was predicted for a woman every 3 months up to age 45 on the basis of the demographic characteristics at that time (age, number of children, and duration since last birth), period set to 2018, certain values of the disorder dummies that we specify below, and the estimated effects of all these factors in the relevant discrete-time hazard model (i.e. age-specific model for first birth and parity-specific models for higher-order births, depending on the number of children already born). We then drew a number from a uniform distribution over [0, 1], and ascribed a singleton birth to the woman within the interval if this number was less than the predicted birth probability. Based on these simulated birth histories, we calculated the average number of children at age 45, the proportion childless at different ages, and the average age at first birth among those who had at least one child at age 45.

In one simulation we set the depression dummy to 1 and the other mental disorder dummies to 0 at all points in time. Although it is uncommon to be constantly depressed, it is not obvious what kind of other depression profile that would be reasonable to choose to illustrate the implications of the estimated effects in the birth-rate models. In the other simulations we set either one of the other mental disorder dummies to 1 and the others to 0, or (to illustrate the implications of having none of the disorders) all of them to 0. We then repeated the procedure for men.

## Results

### Main Patterns

Among women, 5.6% of the time under exposure for a first birth is in the depression category (i.e. during the calendar year before the preceding they had a least one consultation where depression was registered as the diagnosis). The corresponding proportions for anxiety, schizophrenia, bipolar disorder, eating disorder and personality disorder are 2.3%, 0.3% 0.6%, 0.9% and 0.8%, respectively (Table 2). The proportion with schizophrenia is higher among men than women, but all the other disorders are less common among men. The patterns in the distribution of the mental disorders are not very different among individuals under exposure for second or third births, although especially the proportion with schizophrenia is smaller at higher parities, because of the very low first-birth rates (Table 2). See Appendix Table A4.1 in the online supplement for further information about how common the various disorders are.

**Table 2:** Effects (odds ratios with 95% CI) of disorder indicators in discrete-time hazard models for first-, second- and third-birth rates. Norwegian women and men 2010–2018

	First births			Second births			Third births		
Dichotomous disorder indicators	Proportion of exposure time with this disorder (%)	Number of first births among those with this disorder	Effects on first-birth rates ^a^	Proportion of exposure time with this disorder (%)	Number of second births among those with this disorder	Effects on second- birth rates ^b^	Proportion of exposure time with this disorder (%)	Number of third births among those with this disorder	Effects on third-birth rates ^b^
*Panel A: Women*									
Depression	5.59	9161	0.91*** (0.89–0.92)	7.39	6335	0.70*** (0.69–0.72)	5.69	2656	0.87*** (0.83–0.90)
Anxiety	2.28	3883	0.91*** (0.88–0.94)	3.19	2681	0.76*** (0.73–0.79)	2.35	1130	0.89*** (0.83–0.94)
Schizophrenia	0.31	109	0.17*** (0.14–0.20)	0.21	56	0.36*** (0.28–0.47)	0.06	19	0.90 (0.57–1.42)
Bipolar disorder	0.61	908	0.74*** (0.69–0.79)	0.92	572	0.61*** (0.56–0.66)	0.57	217	0.85* (0.74–0.97)
Eating disorder	0.90	1069	0.78*** (0.74–0.83)	0.54	512	0.88** (0.81–0.97)	0.28	183	1.11 (0.96–1.28)
Personality disorder	0.84	1031	0.57*** (0.53–0.60)	1.00	625	0.69*** (0.64–0.75)	0.52	257	0.97 (0.86–1.10)
Number of births			162,065			141,826			54,582
Exposure time (million person- quarters)			13.536			3.784			6.431
Number of individuals who contribute to the exposure time			587,718			268,977			326,463

*Panel B: Men*									
Depression	3.03	3613	0.62*** (0.60–0.64)	3.83	2735	0.62*** (0.60–0.65)	2.81	1272	0.90*** (0.85–0.96)
Anxiety	1.46	1956	0.70*** (0.67–0.74)	1.84	1351	0.67*** (0.64–0.71)	1.18	542	0.88** (0.81–0.96)
Schizophrenia	0.58	125	0.09*** (0.08–0.11)	0.24	57	0.32*** (0.25–0.42)	0.07	13	0.44** (0.26–0.77)
Bipolar disorder	0.38	378	0.50*** (0.46–0.56)	0.46	286	0.69*** (0.61–0.77)	0.32	119	0.83* (0.69–0.99)
Personality disorder	0.51	426	0.45*** (0.40–0.49)	0.57	286	0.55*** (0.49–0.62)	0.30	120	0.84 (0.70–1.01)
Number of births			160,278			134,863			52,778
Exposure time (million person- quarters)			17.303			3.690			5.140
Number of individuals who contribute to the exposure time			708,456			264,835			281,641

^*^p < 0.05; ** p < 0.01; *** p < 0.001

^a^The model also included age and period in one-year categories

^b^The model also included age and period in one-year categories, as well as duration in the following categories (months): < 6, 6–8, 9–11, 12–17, 18–23, 24–29, 30–35, 36–47, 48–59, 60–71, 72–83, 84–95, 96–107, 108–119, and ≥ 120

**Fig. 1:**
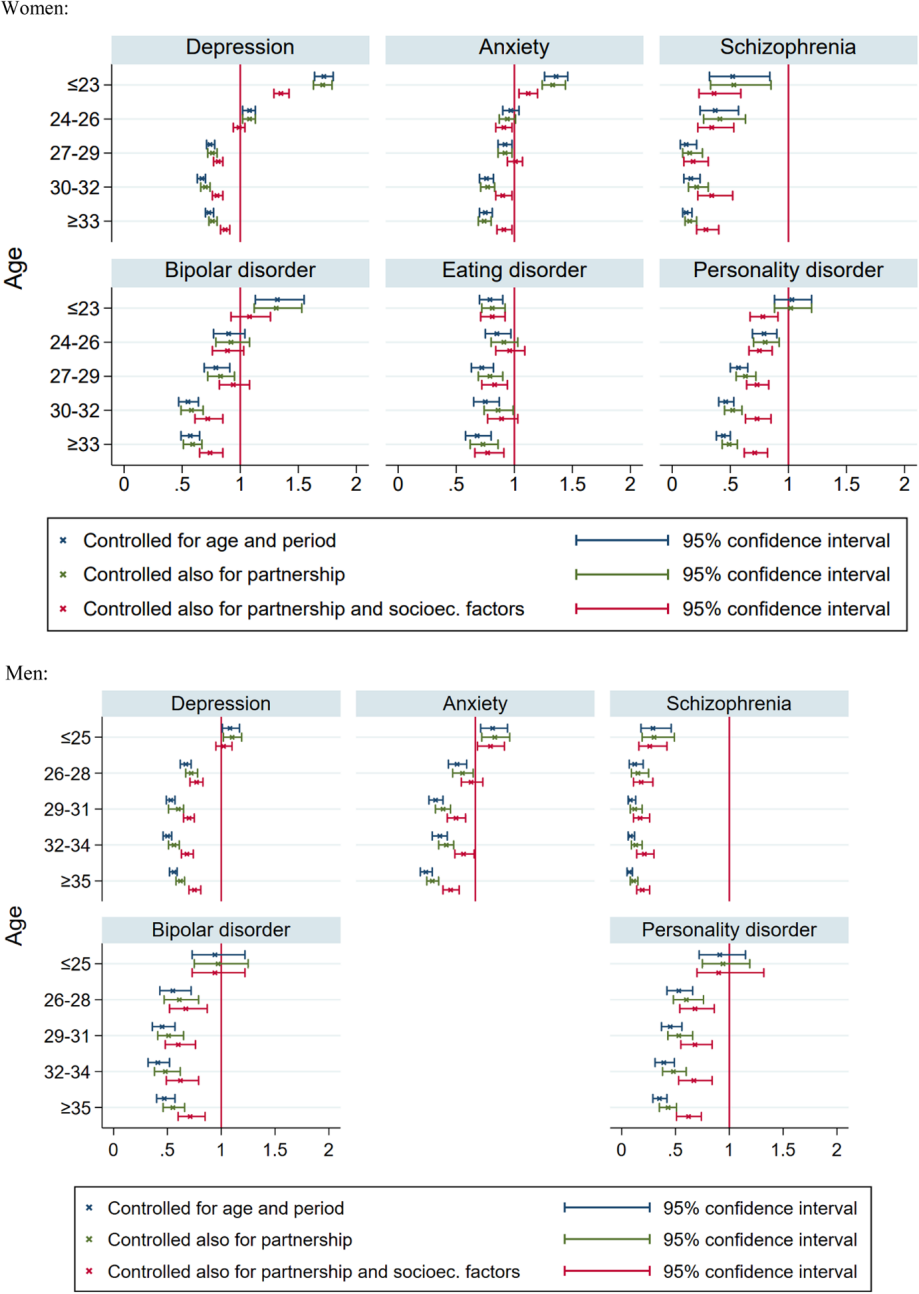
Effects (odds ratios with 95% CI) of disorder indicators in discrete-time hazard models for first-birth rates, by age, according to different models. Norwegian women and men 2010-2018^a^ *Notes*: ^a^Age and period are included with one-year categories. The graphs are based on estimates shown in Appendix Table A6.2 in the online supplement

Depression is associated with a 9% reduction in women’s first-birth rate (Table 2). More specifically, women who had a consultation for depression two years earlier have 9% lower odds of having a first birth than women without such a consultation who are similar with respect to the other variables included in the model (age, year and consultations for other mental disorders, although relatively few women with depression had co-occurring mental disorders). Women with anxiety also have 9% reduced fertility. However, the reductions are limited to the higher age groups; first-birth rates up to age 23 are relatively high among women with these disorders (see the estimates in Fig. 1 that are marked as ‘controlled for age and period’). This means that if entering motherhood at all, they tend to do so relatively early.

Women with bipolar disorder, eating disorder, or personality disorder have even lower first-birth rates, and those with schizophrenia have the lowest. The age difference in the effect coefficients is larger for bipolar disorder and personality disorder than for schizophrenia and eating disorder. Stated differently, it is less clear that women with the latter diseases have a relatively early first birth.

The associations between mental disorders and first births are similar for men, but generally more negative or (at the lowest ages) less clearly positive. There are also negative relationships between mental disorders and second- and third-birth rates, for both sexes (Table 2). Among women, the relationships with depression or anxiety are stronger for second births than first births (all ages pooled); among men, there is little difference. Otherwise, the relationships with mental disorders are weaker for second births than for first births, and even weaker for third births.

Estimates from an analysis of fourth and fifth births, which are quite uncommon, are shown in Appendix Table A4.2 in the online supplement. There are no negative relationships with mental disorders; instead, some estimates point in the opposite direction. Fourth and fifth births were ignored in the remaining analysis, except in the simulations.

### Simulations

The estimated effects on parity- and age-specific birth rates just reported were ‘translated’ into more intuitively informative fertility measures by means of simulation. Note that the underlying assumption in these simulations was that the individuals hypothetically have at least one consultation for, for example, depression every year throughout the reproductive age span, but no consultation for the other disorders. The effects of this depression were set to the estimated effects of depression in year t-2 in the parity- and age-specific models.

For women without any of the mental disorders, the simulated average number of births up to age 45 is 1.60 (Table 3). The number would have been slightly higher if sixth and higher-order births, as well as multiple births, had been included. In comparison, the national total fertility rate for 2018 (including, of course, individuals with mental disorders) was 1.56 (Statistics Norway, 2025a). The simulated childlessness at age 45 is 21.0%, and the average age at first birth among those having become mothers by age 45 is 30.7 years. Official fertility statistics do not include any corresponding ‘synthetic’ numbers based on fertility in 2018 (but include observed childlessness at age 45 in various birth cohorts).

**Table 3:** Simulations of fertility based on estimated effects in birth-rate models

	Average number of children at age 45	Per cent childless at age 25	Per cent childless at age 30	Per cent childless at age 35	Per cent childless at age 45	Average age (in years) at first birth among those who have at least one child at age 45
*Women*						
None of the disorders	1.60	88.1	58.2	32.5	21.0	30.7
Depression	1.34	83.6	60.7	40.4	29.1	30.2
Anxiety	1.39	86.3	59.8	38.4	27.5	30.4
Schizophrenia	0.36	94.6	88.2	81.4	77.3	30.4
Bipolar disorder	1.17	86.9	63.6	45.9	35.7	30.3
Eating disorder	1.35	90.0	66.2	43.5	32.2	31.0
Personality disorder	1.05	89.1	69.9	53.7	44.2	30.6

*Men*						
None of the disorders	1.41	94.2	72.2	46.6	29.6	32.5
Depression	0.90	93.7	79.5	63.3	49.2	32.5
Anxiety	1.01	93.2	76.4	57.7	45.2	32.0
Schizophrenia	0.16	98.3	95.5	92.1	89.2	32.3
Bipolar disorder	0.81	94.3	82.2	67.8	54.9	32.6
Personality disorder	0.71	94.5	82.6	69.3	59.3	32.1

The simulations were based on estimated effects in age-stratified equations for first births (shown in the upper part of the panels for women and men in Appendix Table A6.2 in the online supplement), equations for second and third births (Table 2), and an equation for fourth and fifth birth (Appendix Table A4.3 in the online supplement). Births of sixth or higher order were ignored. All births were assumed to be singleton births. Calendar year was set to 2018 in the simulations. See main text for additional details about the simulation procedure

Among women with depression, the simulated average number of children at age 45 is 1.34 and the proportion childless is 29.1%. The average age at first birth (30.2 years) is slightly lower than for mothers without a mental disorder—as expected from the estimates from the age-stratified models. Furthermore, and also in line with these estimates, the proportion childless at age 25 is actually *lower* than among women without a mental disorder, while the proportion is higher from age 30. The same age pattern in childlessness is seen among those with anxiety, who also end up with almost the same number of children as the depressed.

The simulated average number of children at age 45 among women with schizophrenia is 0.36, while 77.3% are childless. Among women with the other mental disorders, the number of children varies between 1.05 and 1.35. All groups except those with eating disorder have a slightly lower age at first birth than those without a disorder.

Among men without a mental disorder, the simulated fertility at age 45 is 1.41 and childlessness is 29.6%. In comparison, the national total fertility rate in 2018 was 1.41 (Statistics Norway, 2025b), and if the contribution from ages 45–54 was subtracted, it was 1.35. The figures for men with depression are 0.90 children and 49.2% childless, and those for men with schizophrenia are 0.16 children and 89.2% childless. In other words, the difference in the number of children between women without a mental disorder and those with depression is 0.26 (i.e. 1.60 and 1.34, respectively), while the corresponding difference among men is 0.51 (1.41 and 0.90, respectively), which is larger both in absolute and relative sense.

Fathers with anxiety, schizophrenia or personality disorder have slightly lower age at first birth than those without a disorder. As among women, the proportion childless at age 25 (which is very early in men’s reproduction) is lower among those with depression or anxiety than among those without a disorder, while childlessness otherwise is most common among those with one of the disorders.

A similar simulation based on estimates from a model not including the disorder indicators gave a completed fertility of 1.56 for women and 1.37 for men (not shown in tables), which accords well with the national total fertility rates. When we left out 3-month observations where at least one disorder indicator was 1 before the estimation, the corresponding numbers were 1.61 and 1.42 (which are very close to the results for ‘none of the disorders’ reported above). In other words, if all women and men had been exposed—throughout their reproductive period—to the birth rates observed among individuals who did not have a mental disorder two years earlier, rather than to the actual birth rates, completed fertility would have been 0.05 higher for both sexes. Depression and anxiety contribute 70% of this difference (not shown).

### Alternative Disorder Indicators and Multiple Disorders

Although it would seem reasonable to expect a less reduced fertility for individuals in contact with only primary—and not specialised—healthcare, there were only weak indications of such a pattern; the results were quite similar when we used information only about specialised health care (Appendix 5 in the online supplement), which has been the approach in earlier research.

The time lag used for our health indicators matters more: As expected, the relationships with fertility were weaker if we considered the consultations over a period *before* t-2. The lowest fertility was observed among those who had consultations for the mental disorder both in t-2 and earlier (Appendix 5 in the online supplement).

Supplementary analysis also showed that the estimates changed very little when the possibility of multiple disorders in t-2 was taken more explicitly into account (Appendix 5 in the online supplement).

### The Role Played by Partnership, Education, and Income

According to models not stratified by age, the associations between mental disorders and birth rates become somewhat weaker when an indicator of partnership status is added, although almost all the associations that are significant in the simplest model remain significant (Appendix Table A6.1 in the online supplement).

The next step was to also add education level, school enrolment and income to the models. By and large, this further weakened the associations, but less for eating disorder than for the other disorders. Again, almost all associations that were significant according to the simplest model remained significant, the most important exception being the associations between women’s first-birth rates and depression or anxiety. The associations were generally stronger for men than women also with control for these sociodemographic variables. Unemployment, which may both be linked with mental disorder and affect fertility, is partly captured by our categorical income variable. Nevertheless, we estimated supplementary models where we added an indicator of whether the individual experienced unemployment in the year when income was measured. This had no impact on the estimated associations between mental disorders and fertility (tables can be obtained from the authors on request).

Turning to the age-specific models for first births (Fig. 1, based on Appendix Table A6.2 in the online supplement), there are less positive relationships between depression and fertility at low age when all the additional variables are included, while the relationships are less negative at the higher ages (and more because of the socioeconomic factors than partnership). In other words, the tendency among depressed women to become mothers relatively early seems to be partly due to the included sociodemographic variables. A similar pattern appears for the other disorders (except eating disorder), and for both sexes. An attempt to quantify *how much* partnership, education and income (as mediators or selection factors) contribute to the association between mental health and fertility is reported in Appendix 7 in the online supplement.

### Family Fixed-effects Models

When we turned from discrete-time hazard models to Cox models—which is necessary to add family fixed effects—the estimated effects of the disorders were largely unchanged (shown for first births in Appendix Table A6.3 in the online supplement). Leaving out individuals without a same-sex sibling under exposure for the same fertility transition in the study period strongly reduced the sample and made the estimates less precise, but the point estimates of the disorder effects changed little; they were only slightly closer to 1 (Appendix Table A6.4 in the online supplement). This indicates that results from a family fixed-effects analysis based on a ‘sibling sample’ have general relevance.

When family fixed effects were added to the first-birth models (Table 4), the confidence intervals became much broader, and some associations were no longer significant. However, those with depression, schizophrenia, bipolar disorder (only among men), and personality disorder remained significant, and those with anxiety among men and bipolar disorder among women were close to significant (see Appendix Table A6.4 in the online supplement for a comparison with the estimates from the discrete-time model shown in Table 2). After further addition of partnership and socioeconomic factors, the associations with depression among women and personality disorder among men were no longer significant either. In the fixed-effects analysis of second births, only the associations with depression (in both sexes) and anxiety (in women) were significant—and remained significant when the sociodemographic factors were added—while no associations with third-birth rates were significant.

**Table 4:** Effects (hazard ratios with 95% CI) of disorder indicators in sibling-comparison Cox hazard models for first-, second-, and third-birth rates. Norwegian women and men 2010–2018 who have a same-sex sibling under exposure for the same transition^a^

Dichotomous disorder indicators	Effects on first-birth rates	Effects on second-birth rates	Effects on third-birth rates
*Panel A: Women*			
Depression	0.86*** (0.79–0.93)	0.78** (0.66–0.92)	1.00 (0.82–1.23)
Anxiety	1.04 (0.92–1.18)	0.61*** (0.48–0.79)	0.83 (0.62–1.12)
Schizophrenia	0.23*** (0.13–0.41)	0.69 (0.14–3.43)	0.45 (0.10–2.06)
Bipolar disorder	0.79 (0.62–1.02)	0.88 (0.54–1.41)	1.01 (0.55–1.86)
Eating disorder	0.90 (0.73–1.12)	1.01 (0.59–1.74)	0.71 (0.33–1.51)
Personality disorder	0.67*** (0.53–0.84)	0.85 (0.52–1.40)	1.21 (0.63–2.31)
*Control also for partnership status and socioeconomic factors:*			
Depression	0.94 (0.86–1.03)	0.84* (0.71–1.00)	0.99 (0.81–1.22)
Anxiety	1.07 (0.94–1.23)	0.62*** (0.48–0.81)	0.84 (0.62–1.14)
Schizophrenia	0.26*** (0.14–0.49)	0.83 (0.15–4.45)	0.42 (0.09–1.95)
Bipolar disorder	0.80 (0.62–1.05)	0.94 (0.58–1.52)	1.01 (0.55–1.86)
Eating disorder	1.00 (0.79–1.25)	0.99 (0.57–1.73)	0.72 (0.33–1.53)
Personality disorder	0.74* (0.58–0.94)	1.09 (0.65–1.83)	1.26 (0.65–2.43)
Number of births	70,522	40,669	18,609

*Panel B: Men*			
Depression	0.71*** (0.63–0.80)	0.65** (0.50–0.84)	0.85 (0.63–1.14)
Anxiety	0.86 (0.74–1.01)	0.95 (0.65–1.39)	0.88 (0.53–1.46)
Schizophrenia	0.13*** (0.08–0.22)	1.04 (0.36–3.04)	-
Bipolar disorder	0.49*** (0.35–0.69)	0.70 (0.29–1.71)	0.66 (0.25–1.73)
Personality disorder	0.53** (0.38–0.74)	0.47 (0.21–1.04)	1.12 (0.41–3.06)
*Control also for partnership status and socioeconomic factors:*			
Depression	0.84** (0.74–0.96)	0.75* (0.57–0.98)	0.84 (0.62–1.14)
Anxiety	0.98 (0.83–1.17)	1.05 (0.72–1.54)	0.89 (0.53–1.49)
Schizophrenia	0.24*** (0.14–0.39)	1.40 (0.47–4.12)	-
Bipolar disorder	0.60** (0.41–0.88)	0.78 (0.32–1.95)	0.71 (0.27–1.86)
Personality disorder	0.70 (0.48–1.01)	0.50 (0.22–1.12)	1.13 (0.41–3.13)
Number of births	75,318	37,956	16,836

^*^p < 0.05; ** p < 0.01; *** p < 0.001

-could not estimate because of too few observations

^a^The Cox models included year and (if relevant) duration since last previous birth and sociodemographic control variables, with the same categories as in the other analysis (see notes to Table 2 and Appendix Table A6.1 in the online supplement)

## Discussion and Conclusions

### Depression and Anxiety

Our results accord with theoretical ideas and empirical evidence about reduced fertility among men with depression (Golovina et al., 2023; Power et al., 2013) and internalising behaviour more broadly (Jokela et al., 2014; Evensen & Lyngstad, 2020). We find a negative association with depression for all the first three parity transitions in the simplest models, and according to simulations based on these estimates, a hypothetical group of men who have had a consultation for depression each year throughout their reproductive age span on average have only 0.90 children by age 45 (and 49% are childless), as opposed to 1.41 (30% childless) if they have had none of the mental disorders under study.

What can we conclude about the impact on fertility of having shorter spells of depression, which is more common? First, it should be noted that the models on which the simulations are based do not only capture causal effects of mental disorder on fertility, but also various selective influences. We return to that below. Second, the simulated completed fertility would, of course, have been *higher* if we had assumed some years without depression than with our assumption about constant depression (i.e. fewer years during the reproductive period with exposure to a reduced birth rate because of depression). Third—and this contributes in the opposite direction—the simulated fertility under the latter assumption would probably have been even *lower* if we had based it on other effects of depression than those estimated in our main analysis, where the depression indicator refers to the situation two years earlier. Our analysis based on alternative indicators suggests that the fertility among individuals who have been depressed also more than two years earlier—perhaps for several years—likely is much lower. These arguments are, of course, also relevant for the other disorders.

Men’s birth rates are not quite as strongly associated with anxiety as with depression. Turning to the women, most earlier studies, but not all (Kailaheimo-Lönnqvist et al., 2024), have suggested less reduced fertility after a depression or anxiety diagnosis than among men, or no association with fertility at all. This may partly reflect a less negative effect of low education or income among women, or that they have less severe symptoms. Our analysis shows that both depression and anxiety are associated with reduced first-, second-, and third-birth rates also among women. According to the simulations, the number of children among women with depression or anxiety is 1.34 and 1.39, respectively, as opposed to 1.60 among those without a mental disorder. However, this difference is smaller than that among men.

Among women and men who have become parents, the average age at first birth seems to be slightly lower among those with depression or anxiety than among those without a mental disorder. Some earlier studies have suggested a similar pattern (Laursen & Munk-Olsen, 2010; Jokela et al., 2014; Evensen & Lyngstad, 2020; Golovina et al., 2023).

### The Less Common Mental Disorders

As reported also by others (Bundy et al., 2011; Laursen & Munk-Olsen, 2010; Power et al., 2013), individuals with schizophrenia have very low fertility. According to the simulations, women and men with schizophrenia have 0.36 and 0.16 children, respectively, at age 45, and the proportions childless are 77% and 89%. However, among those who become parents, the average age at first birth is relatively low.

Bipolar disorder, eating disorder and personality disorder are also more strongly linked to fertility than are depression and anxiety, but fertility is less markedly reduced than with schizophrenia. The associations are significant both in the models for first births and, to lesser extent, higher-order births in the simplest analysis. Women with bipolar disorder and men with personality disorder seem to have relatively early first births, while women with eating disorder tend to become parents later.

### Control Variables and Possible Reasons for the Remaining Associations

Leaving the timing issue aside for the moment, the main picture is that the mental disorders are associated with reduced fertility. A quite small part of the associations that appear in the simplest models is due to a lower proportion in a union among those with mental disorders. The coefficients become more markedly changed when also education level, school enrolment and income are added. However, almost all relationships that are significant in the simplest models remain significant. Unfortunately, the interpretation is not clear. Partnership, education and income may be joint determinants of mental health and fertility, but they may also be mediators, in which case we are ‘tapping out’ some of the causal effect of mental health on fertility by controlling for them.

Interestingly, the associations tend to be stronger for men than for women also in the more complex models. In other words, the sex differences appearing with the simplest models are not exclusively a result of a possibly less fertility-reducing effect of low education and income among women, or less impact through partnership because of less severe symptoms (which may nevertheless contribute to sex differences through other channels).

When it is also controlled for unobserved characteristics shared by siblings—which should be considered selection factors rather than mediators—the estimates are much less precise and there is weaker evidence of associations between mental health and fertility. However, all disorders except eating disorder are still significantly associated with either first- or second-birth rates for at least one of the sexes. These remaining associations may reflect a variety of causal effects plus (see Sect. 5.5) selection. The causal mechanisms include fears of being too tired to enjoy the parental role, of not being able to care well for the child, or of transmitting the disorder to the child, as discussed above.

With regard to timing differences, one might expect that fewer years of school enrolment among individuals with mental disorder contributes to a relatively early age at first birth among those who become parents. Low income might have a similar effect among women, while the opposite is more likely for men. Our comparison of estimates from models with and without control for these socioeconomic factors indeed supports the idea that low education and income among women with mental disorders contribute to push their age at first birth down (except with respect to eating disorder). The fact that inclusion of these factors has the same impact on the age-specific effect estimates for men suggests that school enrolment plays a larger role as a selection factor or mediator than income. Differences in partnership also contribute to the association between mental health and age at first birth, but far less.

What can explain the more negative associations between mental disorders (except eating disorder) and first-birth rates at the higher ages than at the lower ages that remain after control for the mentioned factors? In theory, one explanation might be that some prefer to have their child relatively early because they suspect further health deterioration. It is also possible that the relatively early birth is a result of a higher probability of unplanned pregnancies, as indicated in some studies (James-Hawkins et al., 2014; Hall et al., 2015). Additionally, the pattern may reflect a severity selection mechanism: As the age increases, those who are still childless may to a larger extent include individuals with a particularly severe version of the disorder. Finally, the observed relationship between mental disorders and first-birth timing may reflect joint determinants (see Sect. 5.5).

The generally less negative associations with mental disorders at higher parities may reflect a severity selection similar to the one just mentioned: Individuals with the least severe symptoms are more likely to have become parents, and consequently be among those under exposure for having a second or higher-order child. Alternatively, the step into parenthood may be seen as a particularly daunting transition, whereas having an additional child involves less challenging life changes.

### Relevance for Fertility at the Aggregate Level

To the extent that the reduced fertility in quarterly observations with at least one nonzero mental disorder indicator is a result of effects of mental disorder on fertility, and not only reflects joint determinants, it would be reasonable to say that a hypothetical elimination of these disorders would increase completed fertility by 0.05. In other words, the disorders seem to contribute quite modestly to the national fertility level. Similarly, a hypothetical doubling of their prevalence over some future years—which is not entirely implausible given the apparent increase in depression and anxiety over recent decades—would reduce fertility by 0.05. However, the current prevalence is probably larger than indicated by our healthcare data. In that case, elimination or doubling would have larger impact, although perhaps not proportionally larger, as fertility may be less affected among those not seeking professional help for their mental problems.

### Strengths and Weaknesses

While there have already been several investigations of fertility among individuals with mental disorders (outside mainstream demography), we have taken an important step forward methodologically by combining (i) the use of high-quality register data, (ii) measurement of mental health before the measurement of fertility, (iii) simulation from the model estimates, and (iv) inclusion of family fixed effects. We have also considered both sexes (not always done), and have broadened the scope compared to most studies by considering both first and higher-order births and allowing the birth rates to vary by age and parity. Additionally, we have assessed whether there are associations above and beyond those due to partnership, education and income. Using information about primary care was an original step, but turned out not to be very important, except that it made the estimates more precise (by increasing the number of individuals with mental disorder) and made the calculation of aggregate-level fertility implications more reasonable.

A weakness in all such research is that several factors that cannot be adequately controlled for may affect mental health as well as the number of children and age at first birth. Our study includes controls for some sociodemographic characteristics of the index person, although these may be causally intermediate rather than selection factors, and controls for family background factors shared by siblings. The remaining associations may partly reflect various characteristics that are unique to each sibling and affecting both health and fertility. For example, personality and certain experiences in young adulthood may have an influence on both mental health and birth rates. Furthermore, being sub- or infecund at a point in time may increase the probability of depression or other mental disorders, if the individual knows about the situation and is sad because they fear they may never be able to have a child. This sub- or infecundity obviously also reduces the probability of later childbirths.

A related problem is that use of healthcare not only reflects a health problem, but also the inclination to consult health personnel for this problem. Use of healthcare may not itself influence fertility, but it is possible that certain characteristics we do not control for both increase the probability of seeking professional help, given the existence of a mental health problem, and reduce the probability of having a child sometime later. Such a pattern would contribute negatively to the estimated associations between mental disorders and subsequent fertility. However, there may also be characteristics operating in the opposite direction, and we are not aware of any evidence suggesting which of the two directions that may be dominating.

The analysis is based on data from a country with an extensive welfare state and a public healthcare system, and it is possible that mental health has a different impact on fertility in other settings. For example, various Norwegian arrangements regarding illness absence from jobs or opportunities to return to school after discontinuation may reduce the impact of mental disorders on income and education, with implications for fertility.

## Supplementary Information

Below is the link to the electronic supplementary material.

Supplementary file1 (DOCX 52 KB)

## Data Availability

The data used in this study are reckoned as highly sensitive. They can only be used in collaboration with the Centre for Fertility and Health at the Norwegian Institute of Public Health, and to examine associations between individuals’ health and factors related to their family situation and reproduction.
