# Supplementary material for: Fertility among Norwegian Women and Men with Mental Disorders
Source: Eur J Popul. 2025 Jul 16;41(1):17. doi: 10.1007/s10680-025-09739-5 (PMC12267794; doi:10.1007/s10680-025-09739-5)
Supplement: Supplementary file 1 — Supplementary file1 (DOCX 52 KB) [file 10680_2025_9739_MOESM1_ESM.docx]

**ONLINE APPENDICES**

**Contents**

**Appendix 1: Additional information about data and methods (with Appendix Table**

**A1.1)**

**Appendix 2: A multilevel-multiprocess model (with Appendix Table A2.1)**

**Appendix 3: A comment on the estimation of sibling models (with Appendix Table A3.1)**

**Appendix 4: Appendix Tables A4.1- A4.2**

**Appendix 5: Alternative disorder indicators and multiple disorders (with Appendix**

**Tables A5.1-A5.4)**

**Appendix 6: Appendix Tables A6.1-A6.4**

**Appendix 7: Quantifying the contribution from partnership, education and income**

**(with Appendix Table A7.1)**

**Appendix 1: Additional information about data and methods**

*Data sources*

Primary healthcare:

Primary health care personnel report consultations to KUHR in order to be reimbursed by the state. Additionally, KUHR includes some consultations with specialists. In the data extracted for the present analysis, 99% of the consultations were with physicians whom it may be reasonable to refer to as general practitioners. The few general practitioners who do not have a contract with the health authorities, and therefore do not benefit from public subsidies, do not report to KUHR.

Specialized health care:

We included consultations (visits) in the following types of specialized health care: somatic hospitals (data available from 2008), private specialists in somatic medicine who had contract with the regional health authorities, and therefore benefitted from public subsidies (‘AVTSOM’, data available from 2009, but a sharp increase in the number of registrations up to 2013 suggests under-registration in 2009-2012), mental hospitals for adults (‘PHV’, data available from 2008), private psychiatrists with adult patients (‘AVTPHV’, data available from 2008), mental hospitals for children and adolescents (‘PHVBU’, data available from 2008), and drug abuse treatment (data available from 2009, although possible under-registration 2009-2010).

In most of these data files, up to two main diagnoses and 20 secondary diagnoses are reported. In other files, there are up to 10 secondary diagnoses (AVTSOM and AVTPHV), or no distinction is made between main and secondary diagnoses (PHVBU). Among the consultations reported in the latter file, 21% did not include any diagnosis at all, while this is uncommon in the other files (0.1% - 5%). For simplicity, we only took into account 10 diagnoses, including all main diagnoses where such diagnoses are defined. There are very few consultations with a larger number of diagnoses reported (e.g., 0.8% of the hospital consultations).

*Restrictions*

Immigrants:

We excluded immigrants because many of them may have children who have never lived in Norway, and who have therefore not been included in the Population Register (Kravdal 2021). This reduced the sample by about 1/5. However, the effects of mental health on fertility did not vary much depending on whether the immigrants were included.

Living in Norway in past years:

The reason for excluding individuals who had not lived in Norway 1^st^ January each year since 2008 up to the quarter in focus was that the number of health care consultations in one of the years back to 2008 (used to construct the key independent variables) is a less informative measure for persons not living in the country the whole year. Living in Norway both at the beginning of a year and the beginning of the next is considered an indication of living in the country the whole year.

*Details about the discrete-time hazard models*

Length of observation intervals:

The fertility data would make it possible to use one-month observation intervals, but we have used three-month intervals (quarters) to simplify data operations and reduce estimation time. These are sufficiently short intervals, because almost identical estimates were obtained with 12-month observation intervals – even for second births. Except for age, calendar year and duration since previous birth, the variables in the model refer to one-year periods or various time points (1^st^ January for partnership status or 1^st^ October for educational level and school enrolment) before the calendar year that includes the quarter.

Multiple births:

In the analysis of first births, both singleton and multiple births were reckoned as first births. However, those who, for example, had a twin first birth did not contribute to the analysis of second births, but to the analysis of third and possibly higher-order births. Multiple births after the first were handled similarly.

The scaling problem:

When effects on fertility are estimated from age-specific logistic models, and these are compared - or fed into simulations - to draw conclusions about fertility timing, some carefulness is warranted. This is because estimates from logistic (as opposed to linear) models for different samples are not fully comparable (Mood 2010). In principle, differences in effect estimates across age may arise just because of age differences in unexplained variance. However, a switch of the sign of the effect, from positive at low age to negative at high age, which appears in parts of our analysis, cannot be explained by such differences in unexplained variance.

*Details about the Cox model*

In the Cox models, age was the underlying time scale, and we assumed that exposures started at the beginning of the relevant quarters and ended (possibly with a birth ‘event’) at the end of the quarters. Period, duration since previous birth and all other variables were included with the same categories as in the discrete-time models.

*Details about included variables*

The importance of controlling for period:

It is potentially important to control for period, because i) unobserved characteristics that change over period are likely to affect fertility and ii) the health indicators are linked to period, partly because of changes in the registration practice (not least the apparently strong reduction in the under-registration of some types of specialized healthcare over the first few years after 2008).

Income:

The income is the sum of labour income, entrepreneurial income, capital income, pensions and some benefits, minus tax deductible amounts.

In the analysis of second and higher-order births, the year t-2 may include a period when one or both parents earned relatively little because they stayed home (full- or part-time) to care for the most recently born child after the paid parental leave period (which most Norwegian parents have). However, for other individuals the income in this year should be a reasonably good indicator of the purchasing power they have had for some time and can expect for some time into the future.

*Analysis alternatives*

Alternatives to the age-stratified analysis of first births:

Instead of doing an age-stratified analysis, one might consider including age interactions. However, there would cause some problems, as interactions on a log-odds or odds scale do not necessarily translate into the same interactions at the probability scale (Ai and Norton 2003).

Lags:

One might consider using longer lags for the sociodemographic variables, so that it would be more reasonable to consider them as selection variables, but this would be far from a perfect solution, because the health indicator based on consultations in t-2 may reflect a health problem that has lasted for some time. Conversely, one might consider including a health indicator for t-3, so that it is more reasonable to consider the sociodemographic variables measured in t-2 as mediators, but the same issue then arises: For example, since earnings after all are relatively stable, a measurement in t-2 also reflects the earnings quite far back in time, which may have influenced the chance of having poor mental health in t-3.

*Additional control variables included in supplementary analysis*

Controls for parental characteristics:

In supplementary analysis, we included several sociodemographic characteristics of the index person’s parents into the discrete-time hazard models (i.e., the models without family fixed effects). This did not affect the key estimates (see Appendix Table A1.1). These characteristics, measured when the index person was 17 years old, were: whether the mother was identified in the data, whether she was alive and resident in Norway, her educational level, her income, her country of birth (Norway vs other), the corresponding variables for the father, and whether the parents were marital or cohabitational partners. Clearly, these characteristics are to a very large extent shared between maternal siblings. This means that the changes in the estimates that we see when family fixed effects are added to the models including mental disorder, age, duration, year, partnership, education and income (see section 4.5) must reflect that there are *other* shared characteristics with an influence on both mental disorders and fertility.

Controls for place of residence:

Because one might also suspect that characteristics of the place of residence are important confounders, we included dummies for the index person’s current municipality of residence in a supplementary analysis where we – to reach convergence within reasonable time – used linear probability models rather than logistic models. Inclusion of these dummies had no impact on the key estimates (not shown in tables).

*References*

Ai, C., & Norton, E.C. (2003). Interaction terms in logit and probit models. *Economics Letters,* 80, 123-129.

Kravdal, Ø. (2021). Sex differences in childlessness in Norway: Identification of underlying demographic drivers. *European Journal of Population,* 37, 1023-1041.

Mood, C. (2010). Logistic regression: Why we cannot do what we think we can do, and what we can do about it. *European Sociological Review,* 26(1), 67-82.

**Appendix Table A1.1. Effects (odds ratios with 95% CI) of disorder indicators in discrete-time hazard models for first-birth rates.** **Norwegian women and men 2010-2018**

|  | Women | | Men | |
| --- | --- | --- | --- | --- |
| Dichotomous disorder indicators | As Table 2 | Also control for family-of-origin characteristics ^a^ | As Table 2 | Also control for family-of-origin characteristics ^a^ |
| Depression | 0.91*** (0.89-0.92) | 0.89*** (0.87-0.91) | 0.62*** (0.60-0.64) | 0.63*** (0.60-0.65) |
| Anxiety | 0.91*** (0.88-0.94) | 0.89*** (0.86-0.92) | 0.70*** (0.67-0.74) | 0.71*** (0.68-0.74) |
| Schizophrenia | 0.17*** (0.14-0.20) | 0.17*** (0.14-0.21) | 0.09*** (0.08-0.11) | 0.09*** (0.08-0.11) |
| Bipolar disorder | 0.74*** (0.69-0.79) | 0.74*** (0.69-0.79) | 0.50*** (0.46-0.56) | 0.51*** (0.46-0.56) |
| Eating disorder | 0.78*** (0.74-0.83) | 0.80*** (0.75-0.85) |  |  |
| Personality disorder | 0.57*** (0.53-0.60) | 0.55*** (0.52-0.59) | 0.45*** (0.40-0.40) | 0.46*** (0.41-0.50) |

*Notes*: *p<0.05; ** p<0.01; *** p<0.001

^a^ The model was as in Table 2, except for the inclusion of the following family-of-origin characteristics: whether the mother was found in the data (yes vs no, in which case she was put in the reference category for the other variables), her country of birth (Norway vs abroad), whether she was alive and living in Norway 1^st^ January the year the child was 17 (yes vs. no, in which case she was put in the reference category for income and partnership), her educational level that year (missing, primary, lower secondary, higher secondary, lower tertiary, higher tertiary, and PhD), her income in NOK that year (missing, 0, 1-49999, 50000-99999, 100000-149999, etc up to 950000-999999, and ≥1000000), corresponding variables for the father, and whether the father and mother were marital or cohabitational partners 1^st^ January the year the child was 17 (that year was before 2005 and the parents were not married with each other, before 2005 and married with each other, 2005+ and neither married nor cohabiting with each other, 2005+ and married with each other, 2005+ and cohabiting with each other).

**Appendix 2: A multilevel-multiprocess model**

As a supplement to the main analysis, we estimated a multilevel-multiprocess model that included seven equations – one for first-birth rates in each of five age groups, one for second-birth rates, and one for third-birth rates. These equations were as in the main analysis, except that the same individual-level normally distributed random term was added to each of them. The model was estimated from a 50% random sample because of constraints in the aML software (Lee and Panis 2003) that was used.

It has been argued that such joint modelling of first-, second-, and third-birth rates may give different estimates of the coefficients (‘effects’) for variables that are strongly linked to the first-birth rates, such as the educational level measured at a relatively high age (Kravdal 2001; Kravdal and Rindfuss 2008). The idea is that individuals have a set of unobserved characteristics with impact on fertility, for example a weak or strong interest in childbearing, or low or high fecundity. Let us, for simplicity, refer to these characteristics as one unobserved fertility determinant – scaled so that a high value of this determinant leads to high fertility. This determinant is represented by the random term, and its value at the start of the reproductive age span is the same, on average, for those who end up with high education and those who end up with low education. However, those among the better educated who ever become parents - and especially those who have their first child early - in spite of all the factors that tend to give the better-educated low fertility, must have higher-than-average value of the unobserved determinant, which also affects later parity transitions positively. That being said, one study showed that joint modelling has little impact on the results when the current (i.e., time-varying) educational level is in focus, rather than the education attained by a high age (Kravdal 2007).

It turned out that the effects of our six time-varying indicators of mental disorder (lagged two years) were unchanged when we estimated a multilevel-multiprocess. This was the case both for women (see Appendix Table A2.1) and men (not shown). The result may reflect that, although the disorders are linked with the chance of having a first child, they are not very strongly associated with the first-birth timing.

*References*

Lillard, L. A., & Panis, C.W.A. (2003). aML Multilevel Multiprocess Statistical Software, Version 2.0. EconWare, Los Angeles, California.

Kravdal, Ø. (2001). The high fertility of college educated women in Norway: An artefact of the separate modelling of each parity transition. *Demographic Research*, 5, 187-216.

Kravdal, Ø. (2007). Effects of current education on second-and third-birth rates among Norwegian women and men born in 1964: Substantive interpretations and methodological issues. *Demographic Research*,17, 211-246.

Kravdal, Ø., & Rindfuss, R.R. (2008). Changing relationships between education and fertility: A study of women and men born 1940 to 1964. *American Sociological Review*, 73(5), 854-873.

**Appendix Table A2.1. Effects (log odds with standard errors) of disorder indicators in discrete-time hazard models for first-, second- and third-birth rates. 50% random sample of Norwegian women 2010-2018**

| Dichotomous disorder indicators | Separate models ^a^ | Joint models ^b^ |
| --- | --- | --- |
| *Effects on first-birth rates, age 17-23* | | |
| Depression | 0.527*** (0.032) | 0.527*** (0.033) |
| Anxiety | 0.355*** (0.052) | 0.356*** (0.052) |
| Schizophrenia | -0.278 (0.303) | -0.278 (0.303) |
| Bipolar disorder | 0.290* (0.115) | 0.290* (0.115) |
| Eating disorder | -0.224* (0.091) | -0.224* (0.091) |
| Personality disorder | -0.039 (0.115) | -0.039 (0.115) |
| *Effects on first-birth rates, age 24-26* | | |
| Depression | 0.105** (0.035) | 0.106** (0.035) |
| Anxiety | -0.068 (0.054) | -0.067 (0.055) |
| Schizophrenia | -0.754** (0.288) | -0.754** (0.288) |
| Bipolar disorder | -0.224 (0.116) | -0.223 (0.117) |
| Eating disorder | -0.137 (0.088) | -0.137 (0.089) |
| Personality disorder | -0.329** (0.102) | -0.328*** (0.102) |
| *Effects on first-birth rates, 27-29* | | |
| Depression | -0.311*** (0.036) | -0.309*** (0.036) |
| Anxiety | -0.165** (0.051) | -0.164** (0.051) |
| Schizophrenia | -1.975*** (0.384) | -1.977*** (0.384) |
| Bipolar disorder | -0.222* (0.098) | -0.222* (0.098) |
| Eating disorder | -0.338*** (0.096) | -0.338*** (0.096) |
| Personality disorder | -0.500*** (0.095) | -0.500*** (0.096) |
| *Effects on first-birth rates, age 30-32* | | |
| Depression | -0.468*** (0.040) | -0.468*** (0.040) |
| Anxiety | -0.343*** (0.056) | -0.344*** (0.057) |
| Schizophrenia | -2.011*** (0.334) | -2.015*** (0.335) |
| Bipolar disorder | -0.407*** (0.108) | -0.408*** (0.108) |
| Eating disorder | -0.235* (0.106) | -0.237* (0.106) |
| Personality disorder | -0.692*** (0.105) | -0.693*** (0.105) |
| *Effects on first-birth rates, age 33-45* | | |
| Depression | -0.364*** (0.036) | -0.365*** (0.036) |
| Anxiety | -0.306*** (0.053) | -0.307*** (0.053) |
| Schizophrenia | -2.118*** (0.237) | -2.123*** (0.237) |
| Bipolar disorder | -0.609*** (0.103) | -0.612*** (0.103) |
| Eating disorder | -0.451*** (0.120) | -0.452*** (0.120) |
| Personality disorder | -0.875*** (0.102) | -0.878*** (0.102) |
| *Effects on second-birth rates* | | |
| Depression | -0.343*** (0.019) | -0.343*** (0.019) |
| Anxiety | -0.263*** (0.028) | -0.263*** (0.028) |
| Schizophrenia | -0.935*** (0.162) | -0.941*** (0.163) |
| Bipolar disorder | -0.493*** (0.058) | -0.495*** (0.059) |
| Eating disorder | -0.140* (0.063) | -0.141* (0.063) |
| Personality disorder | -0.336*** (0.057) | -0.337*** (0.057) |
| *Effects on third-birth rates* | | |
| Depression | -0.215*** (0.030) | -0.215*** (0.030) |
| Anxiety | -0.128** (0.040) | -0.128** (0.044) |
| Schizophrenia | 0.063 (0.309) | 0.061 (0.310) |
| Bipolar disorder | -0.077 (0.091) | -0.077 (0.091) |
| Eating disorder | 0.180 (0.103) | 0.181 (0.103) |
| Personality disorder | 0.026 (0.087) | 0.027 (0.087) |

*Notes*: *p<0.05; ** p<0.01; *** p<0.001

^a^ The models and the samples were as in Table 2 and Figure 1, except for the 50% random selection.

^b^ The model and sample was as for the separate models, except that a random term was added to each of the equations, which were jointly estimated. The estimated standard deviation of the random term was 0.140 (0.030).

**Appendix 3: A comment on the estimation of sibling models**

In principle, sibling model estimates may be biased if an individual’s fertility is influenced by whether the *sibling* has a mental disorder. Such an effect, which may partly reflect that a family member’s mental disorder affects various aspects of the family environment, has indeed been suggested. More specifically, Power et al. (2013) found that sisters of individuals with schizophrenia or bipolar disorder and siblings of individuals with depression or substance abuse had slightly increased fertility, while brothers of individuals with schizophrenia or autism had reduced fertility. Bundy et al. (2011) reported an adverse effect of having a sibling with schizophrenia. However, these associations may reflect not only the mentioned causal effect, but also other mechanisms such as joint social determinants of sibling’s disorder and own fertility or a fear of transmitting genes that may be suspected to be underlying the sibling’s disorder.

If the effect of the sibling’s disease has the same sign as the effect of the person’s own disease (although it likely is considerably weaker), there is a downward bias in the sibling model estimates of the latter effect (Sjölander et al. 2016). To get an impression of the relevance of this issue, we estimated some discrete-time hazard models including indicators of both own and oldest sibling’s mental disorders. The oldest sibling may be a brother or a sister. Control for the sex had no impact on the results. The overall picture is that there are some rather weak associations which go in the same direction as those between fertility and the person’s own mental disorder (shown for first births in Appendix Table A3.1). This indicates that our sibling model estimates may be conservative.

*References*

Bundy, H., Stahl, D., & MacCabe, J.H. (2011). A systematic review and meta‐analysis of the fertility of patients with schizophrenia and their unaffected relatives. *Acta Psychiatrica Scandinavica*, 123(2), 98-106.

Power, R. A., Kyaga, S., Uher, R., MacCabe, J.H., Långström, N., Landen, M., McGuffin, P., Lewis, C.M., Lichtenstein, P., & Svensson, A.C. (2013). Fecundity of patients with schizophrenia, autism, bipolar disorder, depression, anorexia nervosa, or substance abuse vs their unaffected siblings. *JAMA Psychiatry*, 70(1), 22-30.

Sjölander, A., Frisell, T., Kuja-Halkola, R., Öberg, S., & Zetterqvist, J. (2016). Carryover effects in sibling comparison designs. *Epidemiology*, 27(6), 852-858.

**Appendix Table A3.1. Effects (odds ratios with 95% CI) of indicators of own and oldest sibling’s disorders in discrete-time hazard models for first-birth rates. Norwegian women and men 2010-2018 who have at least one sibling ^a^**

| Dichotomous disorder indicators | Women | Men |
| --- | --- | --- |
| *Own disorder* |  |  |
| Depression | 0.91*** (0.88-0.95) | 0.62*** (0.60-0.64) |
| Anxiety | 0.91*** (0.88-0.95) | 0.71*** (0.68-0.74) |
| Schizophrenia | 0.17*** (0.14-0.21) | 0.09*** (0.08-0.11) |
| Bipolar | 0.75*** (0.70-0.80) | 0.51*** (0.46-0.57) |
| Eating disorder | 0.76*** (0.72-0.81) |  |
| Personality disorder | 0.56*** (0.53-0.60) | 0.43*** (0.39-0.48) |
| *Sibling’s disorder* |  |  |
| Depression | 1.01 (0.98-1.04) | 0.95*** (0.93-0.98) |
| Anxiety | 1.02 (0.96-1.06) | 0.95* (0.92-0.99) |
| Schizophrenia | 0.86** (0.78-0.94) | 0.78*** (0.71-0.86) |
| Bipolar disorder | 1.01 (0.93-1.08) | 0.96 (0.89-1.03) |
| Eating disorder | 0.93 (0.85-1.03) |  |
| Personality disorder | 0.93* (0.87-1.00) | 0.90** (0.83-0.96) |
| *Number of births* | 152174 | 151185 |

*Notes*: *p<0.05; ** p<0.01; *** p<0.001

^a^ The models and the samples were as in Table 2, except that those without a sibling were excluded, and a disorder indicator for the sibling (the oldest if there was more than one) was included.

**Appendix 4: Appendix Tables A4.1- A4.2**

**Appendix Table A4.1. Proportion (%) who had at least one consultation in primary or specialized health care for the disorder between 2008 and 2016, by age in 2018 and sex**

| Dichotomous disorder indicators | Women | | Men | |
| --- | --- | --- | --- | --- |
|  | Age 30 | Age 40 | Age 30 | Age 40 |
| Depression | 24.71 | 25.29 | 14.00 | 15.84 |
| Anxiety | 11.87 | 11.83 | 6.82 | 7.64 |
| Schizophrenia | 0.51 | 0.58 | 0.83 | 1.07 |
| Bipolar | 2.14 | 2.25 | 1.25 | 1.49 |
| Eating disorder | 3.11 | 1.42 |  |  |
| Personality disorder | 3.33 | 2.95 | 2.10 | 2.68 |

**Appendix Table A4.2. Effects (odds ratios with 95% CI) of disorder indicators in discrete-time hazard models for fourth- and fifth-birth rates. Norwegian women and men 2010-2018 ^a^**

|  | Women | | | Men | | |
| --- | --- | --- | --- | --- | --- | --- |
| Dichotomous disorder indicators | Propor-tion with this disorder (%) | Number of births among those with this disorder | Effects on birth rates | Propor-tion with this disorder (%) | Number of births among those with this disorder | Effects on birth rates |
| Depression | 5.96 | 889 | 1.15*** (1.07-1.23) | 3.06 | 536 | 1.26*** (1.15-1.38) |
| Anxiety | 2.30 | 358 | 1.08 (0.97-1.20) | 1.12 | 185 | 1.11 (0.95-1.28) |
| Schizophrenia | 0.06 | <10 ^b^ | 0.87 (0.39-1.95) | 0.05 | 10 | 1.31 (0.70-2.45) |
| Bipolar disorder | 0.60 | 96 | 1.20 (0.98-1.48) | 0.37 | 69 | 1.32* (1.04-1.68) |
| Eating disorder | 0.25 | 46 | 1.15 (0.85-1.54) | 0.01 |  |  |
| Personality disorder | 0.54 | 101 | 1.18 (0.96-1.44) | 0.29 | 61 | 1.27 (0.98-1.64) |
| Number of births |  |  | 13007 |  |  | 13781 |

*Notes*: *p<0.05; ** p<0.01; *** p<0.001

^a^ The model also included age and period in one-year categories, parity (3 or 4), and duration as described in Table 2. There were 3.377 million observations among women and 2.504 million among men.

^b^ Numbers below 10 cannot be specified for data protection reasons

**Appendix 5: Alternative disorder indicators and multiple disorders**

*Primary versus specialised care*

As a first step of our analysis of alternative disorder indicators, we checked the implications of considering only specialized health care. We found that, on the whole, those who two years earlier had a consultation for a certain mental disorder in primary health care exclusively had less reduced fertility than those with a consultation for this disorder in specialized health care (some of whom have also had a consultation in primary health care). However, the differences were not large. This is shown for first births in Appendix Table A5.1. Thus, our ability to include consultations in primary health care gives us a larger proportion of individuals in the disorder category under study (making the estimates more precise), but does not have much impact on the point estimates.

*Consultations in earlier years*

While our main focus is on a person’s health care consultations two years earlier (i.e., t-2), we also estimated some models where the key independent variable was whether there had been consultations for the various diseases in at least one year between 2008 and *t-2* or between 2008 and t-3. The use of such an accumulative indicator makes, of course, a control for calendar year particularly important.

As an example of how large the various groups are, let us consider women who are under exposure for a first birth: 5.7% had a consultation for depression in t-2 (3.6% of whom also had such a consultation an earlier year), while 7.9% only had a consultation in an earlier year (not shown). If we focus on those under exposure for a first birth in 2018, 6.2% had a consultation for depression in t-2 (i.e., 2016), while 10.8% only had such a consultation an earlier year (i.e., one of the years 2008-2015, but not 2016). Among the 6.2% who had a consultation in t-2, 4.2% had a consultation also in one of the earlier years. The pattern was similar for anxiety, but the proportion with a consultation for one of the other disorders in 2008-2015, but not in 2016, was smaller than the proportion with a consultation in 2016 – reflecting the more lasting nature of these disorders.

As one might expect, the relationship between fertility and use of health care for mental disorders from 2008 to t-2 was somewhat less negative than that between fertility and the corresponding indicators for t-2 (shown for first births in Appendix Table A5.2). When we included both indicators for 2008 to t-3 and the usual ones for t-2, which would only be meaningful for the years from 2011, most of the coefficients for the latter indicators were stronger than those for the former, but not all of them (Appendix Table A5.3).

In a final step we included indicators of having consultations for the disease both sometime during the period from 2008 to t-3 and in t-2, which indicates a relatively long-lasting disease. As expected, the effect coefficients in these models were particularly strong (for example 0.84 for depression among women as opposed to 0.90 when the focus was on only t-2 (see Model 4 in Appendix Table A5.3).

*Multiple disorders*

Some individuals had consultations for more than one disorder in year t-2. If they, for example, had one consultation for depression and another for eating disorder, or one consultation where both these diagnoses were registered, both the depression indicator and the eating disorder indicator were set to 1 (while the other disorder indicators were 0 if there were no other consultations that year). In order to get more insight into the importance of having multiple disorders (comorbidity), we added an indicator of the number of different mental disorders in t-2 in a supplementary analysis – for simplicity restricted to first births. Among women, the coefficient for depression was then no longer 0.91, as reported earlier, but 0.89 (Appendix Table A5.4). Stated differently, those who had only depression had first birth odds 11% below that of women without any of the mental disorders, rather than 9%, which may be considered as a weighted average of the difference between women with depression and women without depression who have no other disorders and a series of differences between women with depression and women without depression who have a certain other combination of other disorders (many such combinations being possible). This reflects that depression has less impact when the individual also has other disorders.

The comorbidity indicator is positively linked with fertility (Appendix Table A5.4). According to this expanded model, we would predict the odds of a first birth, relative to those without any disorder, as 0.89 ∙ 0.74 ∙ 1.09=0.72 for women with depression and eating disorder (but no other disorder). In other words, having both depression and eating disorder reduces fertility less than one would expect by just combining the reduction due to having only depression (0.89) and the reduction due to having only eating disorder (0.74). However, this total reduction of 0.72 is not very different from what we would predict from our main model for women having depression and eating disorder (but no other disorder): 0.91 ∙ 0.78 = 0.71.

Also the coefficients for the other four disorders become slightly more different from 1 when comorbidity is taken into account in this simple way. Consequently, those with only one disorder would – according to this model - have slightly fewer children than indicated by our simulation. The pattern is similar for men.

Note, however, that it is not very common to be registered with more than one disorder within a year. Among women, 6.7% of the exposure time was with one disorder, 1.5% was with two, and 0.3% was with three or more. The corresponding proportions among men were 4.3%, 0.7%, and 0.1%. However, among women registered with personality disorder, two-thirds also had another disorder, and often depression or anxiety (not shown in tables). In contrast, only one-fourth of those with depression had another disorder, which in more than half of the cases included anxiety.

**Appendix Table A5.1. Effects (odds ratios with 95% CI) of disorder indicators in discrete-time hazard models for first-birth rates. Norwegian women and men 2010-2018 ^a^**

|  | Women | | Men | |
| --- | --- | --- | --- | --- |
| Three-category disorder indicators | Propor-tion of exposure time with this disorder (%) | Effects on first-  birth rates | Propor-tion of exposure time with this disorder (%) | Effects on first-birth rates |
| Depression, only primary care | 3.00 | 0.93*** (0.91-0.96) | 1.84 | 0.64*** (0.61-0.66) |
| Depression, specialized care | 2.59 | 0.87*** (0.84-0.90) | 1.19 | 0.58*** (0.55-0.62) |
| Anxiety, only primary care | 1.18 | 0.91*** (0.87-0.95) | 0.90 | 0.70*** (0.66-0.74) |
| Anxiety, specialized care | 1.10 | 0.91*** (0.87-0.95) | 0.56 | 0.72*** (0.67-0.78) |
| Schizophrenia, only primary care | 0.11 | 0.22*** (0.16-0.29) | 0.19 | 0.10*** (0.08-0.14) |
| Schizophrenia, specialized care | 0.20 | 0.15*** (0.11-0.19) | 0.39 | 0.09*** (0.07-0.11) |
| Bipolar disorder, only primary care | 0.22 | 0.75*** (0.68-0.84) | 0.15 | 0.50*** (0.43-0.59) |
| Bipolar disorder, specialized care | 0.39 | 0.73*** (0.67-0.79) | 0.23 | 0.50*** (0.44-0.58) |
| Eating disorder, only primary care | 0.11 | 0.77** (0.66-0.90) |  |  |
| Eating disorder, specialized care | 0.78 | 0.79*** (0.74-0.85) |  |  |
| Personality disorder, only primary care | 0.12 | 0.55*** (0.46-0.65) | 0.15 | 0.49*** (0.41-0.58) |
| Personality disorder, specialized care | 0.72 | 0.57*** (0.54-0.61) | 0.36 | 0.43*** (0.38-0.48) |

*Notes*: *p<0.05; ** p<0.01; *** p<0.001

^a^ The models were as in Table 2, except for the more detailed disorder indicators. Each of them includes three categories. The category ‘only primary care’ refers to whether there was at least one consultation for the disorder in primary care in the year t-2, but no consultation for the disorder in specialized care. The category ‘specialized care’ refers to whether there was at least one consultation for the disorder in specialized care in the year t-2. There may or may not have been a consultation also in primary care. Those without a consultation for the mental disorder in focus are in the reference category (not shown in the table).

**Appendix Table A5.2. Effects (odds ratios with 95% CI) of disorder indicators in discrete-time hazard models for first-birth rates. Norwegian women and men 2010-2018 ^a^**

| Dichotomous disorder indicators | Women | | Men | |
| --- | --- | --- | --- | --- |
|  | Disorder indicator referring to t-2  (as Table 2) | Disorder indicator referring to the period from 2008 up to t-2 ^a^ | Disorder indicator referring to t-2  (as Table 2) | Disorder indicator referring to the period from 2008 up to t-2 ^a^ |
| Depression | 0.91*** (0.89-0.92) | 0.97*** (0.95-0.98) | 0.62*** (0.60-0.64) | 0.73*** (0.71-0.74) |
| Anxiety | 0.91*** (0.88-0.94) | 0.95*** (0.93-0.97) | 0.70*** (0.67-0.74) | 0.77*** (0.75-0.79) |
| Schizophrenia | 0.17*** (0.14-0.20) | 0.24*** (0.21-0.28) | 0.09*** (0.08-0.11) | 0.17*** (0.15-0.19) |
| Bipolar | 0.74*** (0.69-0.79) | 0.79*** (0.75-0.83) | 0.50*** (0.46-0.56) | 0.65*** (0.61-0.70) |
| Eating disorder | 0.78*** (0.74-0.83) | 0.90*** (0.87-0.94) |  |  |
| Personality disorder | 0.57*** (0.53-0.60) | 0.63*** (0.60-0.66) | 0.45*** (0.40-0.49) | 0.55*** (0.52-0.58) |

*Notes*: *p<0.05; ** p<0.01; *** p<0.001

^a^ The model was as in Table 2, except for the definition of the disorder indicators.

**Appendix Table A5.3. Effects (odds ratios with 95% CI) of disorder indicators in discrete-time hazard models for first-birth rates. Norwegian women and men 2011-2018 ^a^**

Panel A: Women

| Dichotomous disorder indicators | Model 1^a^ | Model 2 ^b^ | Model 3 ^b^ | Model 4 ^b^ |
| --- | --- | --- | --- | --- |
| *Indicators for having the disorder in t-2* |  |  |  |  |
| Depression | 0.90*** (0.88-0.92) |  | 0.92*** (0.90-0.94) |  |
| Anxiety | 0.90*** (0.87-0.93) |  | 0.93*** (0.90-0.97) |  |
| Schizophrenia | 0.17*** (0.14-0.21) |  | 0.38*** (0.30-0.48) |  |
| Bipolar | 0.74*** (0.69-0.80) |  | 0.92* (0.84-0.99) |  |
| Eating disorder | 0.77*** (0.72-0.83) |  | 0.81*** (0.75-0.87) |  |
| Personality disorder | 0.57*** (0.53-0.60) |  | 0.75*** (0.69-0.81) |  |
| *Indicators for having the disorder between 2008 and t-3* |  |  |  |  |
| Depression |  | 0.95*** (0.93-0.97) | 0.98** (0.96-0.99) |  |
| Anxiety |  | 0.94*** (0.92-0.96) | 0.96** (0.94-0.99) |  |
| Schizophrenia |  | 0.24*** (0.21-0.28) | 0.39*** (0.33-0.46) |  |
| Bipolar disorder |  | 0.77*** (0.73-0.81) | 0.81*** (0.75-0.86) |  |
| Eating disorder |  | 0.90*** (0.87-0.94) | 0.97 (0.93-1.02) |  |
| Personality disorder |  | 0.63*** (0.60-0.67) | 0.71*** (0.68-0.76) |  |
| *Indicators of having the disorder both between 2008 and t-3 and in t-2* |  |  |  |  |
| Depression |  |  |  | 0.84*** (0.81-0.87) |
| Anxiety |  |  |  | 0.84*** (0.80-0.88) |
| Schizophrenia |  |  |  | 0.14*** (0.11-0.18) |
| Bipolar disorder |  |  |  | 0.67*** (0.62-0.73) |
| Eating disorder |  |  |  | 0.71*** (0.66-0.77) |
| Personality disorder |  |  |  | 0.54*** (0.50-0.58) |

Panel B: Men

| Dichotomous disorder indicators | Model 1^a^ | Model 2 ^b^ | Model 3 ^b^ | Model 4 ^b^ |
| --- | --- | --- | --- | --- |
| *Indicators for having the disorder in t-2* |  |  |  |  |
| Depression | 0.61*** (0.58-0.63) |  | 0.71*** (0.69-0.74) |  |
| Anxiety | 0.70*** (0.67-0.74) |  | 0.85*** (0.81-0.90) |  |
| Schizophrenia | 0.09*** (0.08-0.11) |  | 0.23*** (0.19-0.28) |  |
| Bipolar | 0.50*** (0.45-0.56) |  | 0.73*** (0.65-0.83) |  |
| Personality disorder | 0.44*** (0.39-0.48) |  | 0.65*** (0.59-0.73) |  |
| *Indicators for having the disorder between 2008 and t-3* |  |  |  |  |
| Depression |  | 0.72*** (0.70-0.73) | 0.78*** (0.76-0.80) |  |
| Anxiety |  | 0.75*** (0.73-0.78) | 0.79*** (0.77-0.82) |  |
| Schizophrenia |  | 0.18*** (0.16-0.20) | 0.36*** (0.32-0.42) |  |
| Bipolar disorder |  | 0.66*** (0.61-0.71) | 0.76*** (0.69-0.83) |  |
| Personality disorder |  | 0.55*** (0.51-0.58) | 0.61*** (0.57-0.65) |  |
| *Indicators of having the disorder both between 2008 and t-3 and in t-2* |  |  |  |  |
| Depression |  |  |  | 0.52*** (0.50-0.55) |
| Anxiety |  |  |  | 0.63*** (0.59-0.67) |
| Schizophrenia |  |  |  | 0.08*** (0.07-0.10) |
| Bipolar disorder |  |  |  | 0.47*** (0.41-0.53) |
| Personality disorder |  |  |  | 0.38*** (0.33-0.44) |

*Notes*: *p<0.05; ** p<0.01; *** p<0.001

^a^ The model was as in Table 2, except for the shorter observation period (2011-2018 rather than 2010-2018)

^b^ The models were as in Table 2, except for the shorter observation period (2011-2018 rather than 2010-2018) and the disorder indicators. Models 2 and 4 included one set of disorder indicators, while Model 3 included two sets of disorder indicators.

**Appendix Table A5.4. Effects (odds ratios with 95% CI) of disorder indicators in discrete-time hazard models for first-birth rates. Norwegian women and men 2010-2018**

|  | Women | | Men | |
| --- | --- | --- | --- | --- |
| Dichotomous indicators for disorder in t-2 | As in Table 2 | As in Table 2, but number of disorders added | As Table 2 | As Table 2, but number of disorders added |
| Depression | 0.91*** (0.89-0.92) | 0.89*** (0.87-0.92) | 0.62*** (0.60-0.64) | 0.58*** (0.56-0.60) |
| Anxiety | 0.91*** (0.88-0.94) | 0.88*** (0.84-0.91) | 0.70*** (0.67-0.74) | 0.64*** (0.60-0.67) |
| Schizophrenia | 0.17*** (0.14-0.20) | 0.17*** (0.14-0.20) | 0.09*** (0.08-0.11) | 0.09*** (0.07-0.10) |
| Bipolar | 0.74*** (0.69-0.79) | 0.70*** (0.66-0.75) | 0.50* (0.46-0.56) | 0.44*** (0.40-0.49) |
| Eating disorder | 0.78*** (0.74-0.83) | 0.74*** (0.70-0.79) |  |  |
| Personality disorder | 0.57*** (0.53-0.60) | 0.52*** (0.49-0.56) | 0.45*** (0.40-0.49) | 0.36*** (0.33-0.40) |
| Number of disorders in  t-2 |  |  |  |  |
| 0 or 1 (reference) |  | 1 |  | 1 |
| 2 |  | 1.09** (1.03-1.16) |  | 1.52*** (1.38-1.68) |
| ≥3 |  | 1.38*** (1.21-1.58) |  | 2.96*** (2.28-3.84) |

*Notes*: *p<0.05; ** p<0.01; *** p<0.001

^a^ Among women, the proportions having 0,1,2 or ≥ 3 number of disorders were 91.5%, 6.7%, 1.5%, and 0.3%, respectively. The corresponding proportions among men were 94.9%, 4.3%, 0.7% and 0.1%.

**Appendix 6: Appendix Tables A6.1- A6.4**

**Appendix Table A6.1. Effects (odds ratios with 95% CI) of disorder indicators in discrete-time hazard models for first-, second- and third-birth rates. Norwegian women and men 2010-2018** **^a^**

Panel A: Women

| Dichotomous disorder indicators | Effects on first-birth rates | Effects on second-birth rates | Effects on third-birth rates |
| --- | --- | --- | --- |
| Depression | 0.91*** (0.89-0.92) | 0.70*** (0.69-0.72) | 0.87*** (0.83-0.90) |
| Anxiety | 0.90*** (0.88-0.94) | 0.76*** (0.73-0.79) | 0.89*** (0.83-0.94) |
| Schizophrenia | 0.17*** (0.14-0.20) | 0.36*** (0.28-0.47) | 0.90 (0.57-1.42) |
| Bipolar disorder | 0.74*** (0.69-0.79) | 0.61*** (0.56-0.66) | 0.85* (0.74-0.97) |
| Eating disorder | 0.78*** (0.74-0.83) | 0.88** (0.81-0.97) | 1.11 (0.96-1.28) |
| Personality disorder | 0.57*** (0.53-0.60) | 0.69*** (0.64-0.75) | 0.97 (0.86-1.10) |
| Number of births | 162062 | 141826 | 54582 |
| Exposure time (million person- quarters) | 13.536 | 3.784 | 6.431 |
| Control also for partnership status: | | | |
| Depression | 0.93*** (0.91-0.95) | 0.75*** (0.73-0.77) | 0.86*** (0.83-0.90) |
| Anxiety | 0.90*** (0.87-0.93) | 0.78*** (0.75-0.81) | 0.89*** (0.83-0.94) |
| Schizophrenia | 0.21*** (0.18-0.26) | 0.44*** (0.34-0.58) | 0.90 (0.57-1.42) |
| Bipolar disorder | 0.76*** (0.71-0.81) | 0.63*** (0.58-0.69) | 0.84** (0.73-0.96) |
| Eating disorder | 0.84*** (0.79-0.90) | 0.93 (0.85-1.01) | 1.10 (0.94-1.27) |
| Personality disorder | 0.62*** (0.58-0.66) | 0.76*** (0.70-0.83) | 0.97 (0.85-1.10) |
| Control also for partnership status and socioeconomic factors: | | | |
| Depression | 0.98 (0.96-1.00) | 0.79*** (0.77-0.82) | 0.90*** (0.86-0.93) |
| Anxiety | 0.98 (0.95-1.01) | 0.85*** (0.81-0.88) | 0.92** (0.87-0.98) |
| Schizophrenia | 0.26*** (0.22-0.32) | 0.53*** (0.40-0.69) | 0.98 (0.62-1.54) |
| Bipolar disorder | 0.86*** (0.81-0.92) | 0.69*** (0.63-0.75) | 0.86* (0.75-0.98) |
| Eating disorder | 0.88*** (0.83-0.93) | 0.95 (0.87-1.04) | 1.11 (0.95-1.28) |
| Personality disorder | 0.73*** (0.68-0.77) | 0.88*** (0.81-0.95) | 1.02 (0.90-1.16) |

Panel B: Men

| Dichotomous disorder indicators | Effects on first-birth rates | Effects on second-birth rates | Effects on third-birth rates |
| --- | --- | --- | --- |
| Depression | 0.62*** (0.60-0.64) | 0.62*** (0.60-0.65) | 0.90*** (0.85-0.96) |
| Anxiety | 0.70*** (0.67-0.74) | 0.67*** (0.64-0.71) | 0.88** (0.81-0.96) |
| Schizophrenia | 0.09*** (0.08-0.11) | 0.32*** (0.25-0.42) | 0.44** (0.26-0.77) |
| Bipolar disorder | 0.50*** (0.46-0.56) | 0.69*** (0.61-0.77) | 0.83* (0.69-0.99) |
| Personality disorder | 0.45*** (0.40-0.49) | 0.55*** (0.48-0.62) | 0.84 (0.70-1.01) |
| Number of births | 160278 | 134863 | 52778 |
| Exposure time (million person- quarters) | 17.303 | 3.690 | 5.140 |
| Control also for partnership status: | | | |
| Depression | 0.68*** (0.66-0.71) | 0.70*** (0.67-0.73) | 0.89*** (0.84-0.94) |
| Anxiety | 0.76*** (0.73-0.80) | 0.72*** (0.68-0.76) | 0.88** (0.81-0.96) |
| Schizophrenia | 0.13*** (0.11-0.16) | 0.39*** (0.30-0.51) | 0.42** (0.24-0.73) |
| Bipolar disorder | 0.57*** (0.52-0.63) | 0.73*** (0.65-0.82) | 0.80* (0.67-0.96) |
| Personality disorder | 0.53*** (0.48-0.58) | 0.63*** (0.56-0.71) | 0.82* (0.68-0.99) |
| Control also for partnership status and socioeconomic factors: | | | |
| Depression | 0.78*** (0.76-0.82) | 0.77*** (0.74-0.80) | 0.90*** (0.85-0.95) |
| Anxiety | 0.90*** (0.86-0.94) | 0.80*** (0.75-0.84) | 0.89** (0.81-0.97) |
| Schizophrenia | 0.19*** (0.16-0.22) | 0.47*** (0.36-0.61) | 0.42** (0.24-0.73) |
| Bipolar disorder | 0.70*** (0.63-0.77) | 0.80*** (0.71-0.90) | 0.80* (0.66-0.96) |
| Personality disorder | 0.68*** (0.61-0.74) | 0.74*** (0.65-0.83) | 0.82* (0.68-0.99) |

*Notes*: *p<0.05; ** p<0.01; *** p<0.001

^a^ The estimates in the upper part of each panel are as in Table 2. All models included age, period and duration as described in Table 2. Some of them also included partnership status (married, widowed and not cohabiting, divorced and not cohabiting, separated and not cohabiting, never-married and not cohabiting, and cohabiting), and some additionally included the following sociodemographic factors: educational level (missing, primary or not completed higher secondary, higher secondary, lower tertiary, and higher tertiary), school enrolment (yes vs. no) and income in NOK, with the following categorization: missing, 0, 1-49999, 50000-99999, 100000-149999, etc up to 950000-999999, and ≥1000000.

**Appendix Table A6.2. Effects (odds ratios with 95% CI) of disorder indicators in discrete-time hazard models for first-birth rates, by age. Norwegian women and men 2010-2018 ^a^**

Panel A: Women

| Dichotomous disorder indicators | Age ≤ 23 | Age 24-26 | Age 27-29 | Age 30-32 | Age ≥ 33 |
| --- | --- | --- | --- | --- | --- |
| Depression | 1.72*** (1.64-1.80) | 1.08** (1.02-1.13) | 0.74*** (0.71-0.78) | 0.67*** (0.63-0.70) | 0.73*** (0.70-0.77) |
| Anxiety | 1.36*** (1.26-1.46) | 0.97 (0.90-1.04) | 0.92* (0.86-0.98) | 0.76*** (0.70-0.82) | 0.75*** (0.70-0.81) |
| Schizophrenia | 0.52*** (0.32-0.84) | 0.37*** (0.24-0.57) | 0.12*** (0.07-0.21) | 0.16*** (0.10-0.24) | 0.12*** (0.09-0.17) |
| Bipolar disorder | 1.32*** (1.13-1.55) | 0.90 (0.77-1.04) | 0.79*** (0.69-0.91) | 0.55*** (0.47-0.64) | 0.57*** (0.49-0.65) |
| Eating disorder | 0.79*** (0.70-0.90) | 0.85* (0.75-0.97) | 0.72*** (0.63-0.82) | 0.75*** (0.65-0.87) | 0.68*** (0.58-0.80) |
| Personality disorder | 1.03 (0.88-1.20) | 0.79*** (0.69-0.90) | 0.57*** (0.50-0.65) | 0.46*** (0.40-0.53) | 0.44*** (0.38-0.50) |
| Number of first births | 26976 | 31583 | 38949 | 32396 | 32161 |
| Control also for partnership status | | | | | |
| Depression | 1.71*** (1.63-1.79) | 1.08** (1.02-1.13) | 0.76*** (0.72-0.80) | 0.70*** (0.66-0.74) | 0.76*** (0.73-0.80) |
| Anxiety | 1.33*** (1.24-1.44) | 0.94 (0.87-1.01) | 0.92* (0.86-0.98) | 0.77*** (0.71-0.83) | 0.74*** (0.69-0.80) |
| Schizophrenia | 0.53*** (0.33-0.85) | 0.41*** (0.27-0.63) | 0.15*** (0.09-0.26) | 0.21*** (0.14-0.31) | 0.15*** (0.11-0.21) |
| Bipolar disorder | 1.31*** (1.12-1.53) | 0.92 (0.79-1.08) | 0.83** (0.72-0.95) | 0.58*** (0.49-0.68) | 0.59*** (0.51-0.67) |
| Eating disorder | 0.81** (0.72-0.92) | 0.91 (0.80-1.03) | 0.79*** (0.69-0.90) | 0.86* (0.74-0.99) | 0.73*** (0.62-0.86) |
| Personality disorder | 1.02 (0.88-1.20) | 0.80*** (0.70-0.92) | 0.63*** (0.55-0.72) | 0.52*** (0.45-0.60) | 0.49*** (0.43-0.56) |
| Control also for partnership status and socioeconomic factors | | | | | |
| Depression | 1.35*** (1.29-1.42) | 0.99 (0.94-1.04) | 0.81*** (0.77-0.85) | 0.80*** (0.76-0.85) | 0.87*** (0.83-0.91) |
| Anxiety | 1.16** (1.12-1.20) | 0.91* (0.84-0.98) | 1.01 (0.94-1.07) | 0.90*** (0.84-0.98) | 0.91* (0.85-0.98) |
| Schizophrenia | 0.36*** (0.23-0.59) | 0.34*** (0.22-0.53) | 0.18*** (0.10-0.31) | 0.34*** (0.22-0.52) | 0.29*** (0.21-0.40) |
| Bipolar disorder | 1.08 (0.92-1.26) | 0.89 (0.76-1.03) | 0.94 (0.82-1.08) | 0.72*** (0.61-0.85) | 0.74*** (0.65-0.85) |
| Eating disorder | 0.81** (0.71-0.92) | 0.96 (0.84-1.09) | 0.83** (0.72-0.94) | 0.89 (0.77-1.03) | 0.77** (0.66-0.91) |
| Personality disorder | 0.78** (0.67-0.91) | 0.75*** (0.66-0.86) | 0.73*** (0.64-0.83) | 0.73*** (0.63-0.85) | 0.71*** (0.62-0.82) |

Panel B: Men

| Dichotomous disorder indicators | Age ≤ 25 | Age 26-28 | Age 29-31 | Age 32-34 | Age ≥ 35 |
| --- | --- | --- | --- | --- | --- |
| Depression | 1.08* (1.01-1.17) | 0.67*** (0.62-0.72) | 0.53*** (0.49-0.57) | 0.50*** (0.46-0.54) | 0.56*** (0.52-0.59) |
| Anxiety | 1.16** (1.05-1.30) | 0.83*** (0.75-0.92) | 0.63*** (0.57-0.70) | 0.67*** (0.60-0.74) | 0.54*** (0.49-0.60) |
| Schizophrenia | 0.29*** (0.18-0.46) | 0.12*** (0.07-0.20) | 0.08*** (0.06-0.13) | 0.08*** (0.06-0.12) | 0.07*** (0.05-0.10) |
| Bipolar disorder | 0.94 (0.73-1.22) | 0.55*** (0.43-0.72) | 0.45*** (0.36-0.57) | 0.41*** (0.32-0.52) | 0.47*** (0.40-0.57) |
| Personality disorder | 0.91 (0.72-1.15) | 0.53*** (0.42-0.66) | 0.45*** (0.37-0.56) | 0.39*** (0.31-0.49) | 0.35*** (0.29-0.42) |
| Number of first births | 26899 | 31613 | 36879 | 28948 | 35940 |
| Control also for partnership status | | | | | |
| Depression | 1.10* (1.02-1.19) | 0.72*** (0.67-0.78) | 0.60*** (0.51-0.65) | 0.56*** (0.51-0.61) | 0.62*** (0.58-0.66) |
| Anxiety | 1.18** (1.06-1.32) | 0.88* (0.79-0.98) | 0.70*** (0.63-0.77) | 0.73*** (0.66-0.80) | 0.60*** (0.55-0.66) |
| Schizophrenia | 0.30*** (0.19-0.49) | 0.15*** (0.09-0.25) | 0.12*** (0.08-0.19) | 0.13*** (0.09-0.19) | 0.11*** (0.08-0.15) |
| Bipolar disorder | 0.97 (0.75-1.25) | 0.61*** (0.47-0.79) | 0.51*** (0.41-0.65) | 0.48*** (0.38-0.62) | 0.55*** (0.46-0.66) |
| Personality disorder | 0.94 (0.75-1.19) | 0.60*** (0.48-0.76) | 0.53*** (0.43-0.66) | 0.48*** (0.38-0.60) | 0.43*** (0.35-0.51) |
| Control also for partnership status and socioeconomic factors | | | | | |
| Depression | 1.02 (0.95-1.10) | 0.77*** (0.71-0.83) | 0.70*** (0.65-0.75) | 0.68*** (0.63-0.74) | 0.75*** (0.70-0.81) |
| Anxiety | 1.14* (1.02-1.27) | 0.96 (0.87-1.07) | 0.82*** (0.74-0.91) | 0.89* (0.81-0.99) | 0.77*** (0.70-0.85) |
| Schizophrenia | 0.26*** (0.16-0.42) | 0.18*** (0.11-0.29) | 0.17*** (0.11-0.26) | 0.21*** (0.14-0.30) | 0.19*** (0.14-0.26) |
| Bipolar disorder | 0.94 (0.73-1.22) | 0.67** (0.52-0.87) | 0.60*** (0.48-0.76) | 0.62*** (0.49-0.79) | 0.71*** (0.60-0.85) |
| Personality disorder | 0.90 (0.70-1.32) | 0.68*** (0.54-0.86) | 0.68*** (0.55-0.84) | 0.67*** (0.53-0.84) | 0.62*** (0.51-0.74) |

*Notes*: *p<0.05; ** p<0.01; *** p<0.001

^a^ All models included age and period with one-year categories. Some of the models also included partnership status (married, widowed and not cohabiting, divorced and not cohabiting, separated and not cohabiting, never-married and not cohabiting, and cohabiting), and some additionally included the following sociodemographic factors: educational level (missing, primary or not completed higher secondary, higher secondary, lower tertiary, and higher tertiary), school enrolment (yes vs. no) and income in NOK, with the following categorization: missing, 0, 1-49999, 50000-99999, 100000-149999, etc up to 950000-999999, and ≥1000000.

**Appendix Table A6.3. Effects (odds ratios or hazard ratios with 95% CI) of disorder indicators in discrete-time hazard models or Cox models for first-birth rates. Norwegian women and men 2010-2018**

Panel A: Full sample ^a^

| Dichotomous disorder indicators | Women | | Men | |
| --- | --- | --- | --- | --- |
|  | Discrete-time hazard model  (as Table 2) | Cox model | Discrete-time hazard model  (as Table 2) | Cox model |
| Depression | 0.91*** (0.89-0.92) | 0.91*** (0.89-0.93) | 0.62*** (0.60-0.64) | 0.62*** (0.60-0.64) |
| Anxiety | 0.91*** (0.88-0.94) | 0.91*** (0.88-0.94) | 0.70*** (0.67-0.74) | 0.71*** (0.67-0.74) |
| Schizophrenia | 0.17*** (0.14-0.20) | 0.17*** (0.14-0.21) | 0.09*** (0.08-0.11) | 0.09*** (0.08-0.11) |
| Bipolar disorder | 0.74*** (0.69-0.79) | 0.74*** (0.70-0.79) | 0.50*** (0.46-0.56) | 0.51*** (0.46-0.56) |
| Eating disorder | 0.78*** (0.74-0.83) | 0.79*** (0.74-0.84) |  |  |
| Personality disorder | 0.57*** (0.53-0.60) | 0.57*** (0.54-0.61) | 0.45*** (0.40-0.49) | 0.45*** (0.41-0.49) |
| Number of births | 162065 | 162065 | 160278 | 160278 |

Panel B: Including only those who have a same-sex sibling ^b^

| Dichotomous disorder indicators | Women | | Men | |
| --- | --- | --- | --- | --- |
|  | Discrete-time hazard model | Cox model | Discrete-time hazard model | Cox model |
| Depression | 0.93*** (0.90-0.96) | 0.93*** (0.90-0.96) | 0.63*** (0.60-0.66) | 0.63*** (0.60-0.67) |
| Anxiety | 0.94* (0.89-0.99) | 0.94* (0.89-0.99) | 0.75*** (0.70-0.80) | 0.75*** (0.70-0.80) |
| Schizophrenia | 0.18*** (0.13-0.24) | 0.17*** (0.13-0.24) | 0.09*** (0.07-0.12) | 0.09*** (0.07-0.12) |
| Bipolar disorder | 0.77*** (0.69-0.85) | 0.77*** (0.69-0.85) | 0.51*** (0.44-0.60) | 0.51*** (0.44-0.60) |
| Eating disorder | 0.77*** (0.70-0.84) | 0.78*** (0.71-0.85) |  |  |
| Personality disorder | 0.59*** (0.54-0.65) | 0.60*** (0.54-0.66) | 0.43*** (0.37-0.49) | 0.43*** (0.37-0.50) |
| Number of births | 70522 | 70522 | 75318 | 75318 |

*Notes*: *p<0.05; ** p<0.01; *** p<0.001

^a^ The models and the samples were as in Table 2. The Cox models included year, but not (unlike the discrete-time hazard models) age, which was the ‘duration variable’.

^b^ The models were as those in Panel A, except that individuals who did not have a same-sex sibling who was also under exposure for first birth were excluded.

**Appendix Table A6.4. Effects (hazard ratios with 95% CI) of disorder indicators in discrete-time hazard models or sibling-comparison Cox models for first-, second-, and third-birth rates. Norwegian women and men 2010-2018**

Panel A: Women

| Dichotomous disorder indicators | Effects on first-birth rates | Effects on second-birth rates | Effects on third-birth rates |
| --- | --- | --- | --- |
|  | | | |
|  | Discrete-time hazard models (estimates copied from Table 2) | | |
| Depression | 0.91*** (0.89-0.92) | 0.70*** (0.69-0.72) | 0.87*** (0.83-0.90) |
| Anxiety | 0.91 (0.88-0.94) | 0.76*** (0.73-0.79) | 0.89*** (0.83-0.94) |
| Schizophrenia | 0.17*** (0.14-0.20) | 0.36*** (0.28-0.47) | 0.90 (0.57-1.42) |
| Bipolar disorder | 0.74*** (0.69-0.79) | 0.61*** (0.56-0.66) | 0.85* (0.74-0.97) |
| Eating disorder | 0.78*** (0.74-0.83) | 0.88** (0.81-0.97) | 1.11 (0.96-1.28) |
| Personality disorder | 0.57*** (0.53-0.60) | 0.69*** (0.64-0.75) | 0.97 (0.86-1.10) |
|  | | | |
|  | Sibling-comparison Cox models (estimates copied from Table 4) | | |
| Depression | 0.86*** (0.79-0.93) | 0.78** (0.66-0.92) | 1.00 (0.82-1.23) |
| Anxiety | 1.04 (0.92-1.18) | 0.61*** (0.48-0.79) | 0.83 (0.62-1.12) |
| Schizophrenia | 0.23*** (0.13-0.41) | 0.69 (0.14-3.43) | 0.45 (0.10-2.06) |
| Bipolar disorder | 0.79 (0.62-1.02) | 0.88 (0.54-1.41) | 1.01 (0.55-1.86) |
| Eating disorder | 0.90 (0.73-1.12) | 1.01 (0.59-1.74) | 0.71 (0.33-1.51) |
| Personality disorder | 0.67*** (0.53-0.84) | 0.85 (0.52-1.40) | 1.21 (0.63-2.31) |

Panel B: Men

| Dichotomous disorder indicators | Effects on first-birth rates | Effects on second-birth rates | Effects on third-birth rates |
| --- | --- | --- | --- |
|  | | | |
|  | Discrete-time hazard models (estimates copied from Table 2) | | |
| Depression | 0.62*** (0.60-0.64) | 0.62*** (0.60-0.65) | 0.90*** (0.85-0.96) |
| Anxiety | 0.70*** (0.67-0.74) | 0.67*** (0.64-0.71) | 0.88** (0.81-0.96) |
| Schizophrenia | 0.09*** (0.08-0.11) | 0.32*** (0.25-0.42) | 0.44** (0.26-0.77) |
| Bipolar disorder | 0.50*** (0.46-0.56) | 0.69*** (0.61-0.77) | 0.83* (0.69-0.99) |
| Personality disorder | 0.45*** (0.40-0.49) | 0.55*** (0.49-0.62) | 0.84 (0.70-1.01) |
|  | | | |
|  | Sibling-comparison Cox models (estimates copied from Table 4) | | |
| Depression | 0.71*** (0.63-0.80) | 0.65** (0.50-0.84) | 0.85 (0.63-1.14) |
| Anxiety | 0.86 (0.74-1.01) | 0.95 (0.65-1.39) | 0.88 (0.53-1.46) |
| Schizophrenia | 0.13*** (0.08-0.22) | 1.04 (0.36-3.04) | - |
| Bipolar disorder | 0.49*** (0.35-0.69) | 0.70 (0.29-1.71) | 0.66 (0.25-1.73) |
| Personality disorder | 0.53** (0.38-0.74) | 0.47 (0.21-1.04) | 1.12 (0.41-3.06) |

*Notes*: *p<0.05; ** p<0.01; *** p<0.001

-could not estimate because of too few observations

**Appendix 7: Quantifying the contribution from partnership, education and income**

It is difficult to quantify how much partnership, education, and income contribute (as mediators or selection factors) to the associations between mental health and fertility. This is because of a ‘scaling problem’ that arises in logistic models (Mood 2010). The essence of that problem is that effect coefficients for variables V in logistic models may become stronger even when variables uncorrelated with V are added. However, by comparing estimates from models with and without these factors one gets at least a conservative measure of their contribution.

Starting with bipolar disorder as a first example, the coefficient in the model for women’s first birth changes from 0.74 (i.e., the odds of a first birth are reduced by 26%) in the simplest model, to 0.76 when partnership is controlled for, and to 0.86 (i.e. odds reduced by 14%) when also the socioeconomic indicators are added. However, one cannot conclude that these variables in total account for 46% of the association (1-0.14/0.26), given the ‘scaling problem’. Their contribution is somewhat larger. As an alternative assessment, we estimated corresponding linear probability models, where there is not a similar scaling problem (and which involve additive rather than multiplicative effects on fertility). Inclusion of sociodemographic variables in these models reduced the association by 56% (Appendix Table A7.1). Similar calculations for other disorders and parity transitions, and for both sexes, show that the reduction in the effect coefficients varies between 33% and 77%.

*Reference*

Mood, C. (2010). Logistic regression: Why we cannot do what we think we can do, and what we can do about it. *European Sociological Review*, 26(1), 67-82.

**Appendix Table A7.1. Effects (linear effects with standard errors) of disorder indicators in discrete-time hazard models for first-birth rates, when linear probability models are estimated from the 3-month observations. Norwegian women and men 2010-2018 ^a^**

Panel A: Women

| Dichotomous disorder indicators | Effects on first-birth rates | Effects on second-birth rates | Effects on third-birth  rates |
| --- | --- | --- | --- |
| Depression | -0.0012*** (0.0001) | -0.0092*** (0.0004) | -0.0011*** (0.0002) |
| Anxiety | -0.0013*** (0.0002) | -0.0072*** (0.0006) | -0.0010*** (0.0002) |
| Schizophrenia | -0.0126*** (0.0005) | -0.0119*** (0.0021) | -0.0005 (0.0014) |
| Bipolar disorder | -0.0041*** (0.0004) | -0.0102*** (0.0010) | -0.0012* (0.0005) |
| Eating disorder | -0.0024*** (0.0003) | -0.0036*** (0.0013) | 0.0007 (0.0007) |
| Personality disorder | -0.0073*** (0.0003) | -0.0074*** (0.0010) | -0.0005 (0.0005) |
| Control also for partnership status and socioeconomic factors: | | | |
| Depression | -0.0004** (0.0001) | -0.0058*** (0.0004) | -0.0009*** (0.0002) |
| Anxiety | -0.0003 (0.0002) | -0.0036*** (0.0006) | -0.0007** (0.0002) |
| Schizophrenia | -0.0046*** (0.0005) | -0.0012 (0.0021) | -0.0001 (0.0014) |
| Bipolar disorder | -0.0017*** (0.0004) | -0.0067*** (0.0010) | -0.0011* (0.0005) |
| Eating disorder | -0.0014*** (0.0003) | -0.0018 (0.0013) | 0.0007 (0.0007) |
| Personality disorder | -0.0032*** (0.0003) | -0.0010 (0.0010) | -0.0001 (0.0005) |

Panel A: Men

| Dichotomous disorder indicators | Effects on first-birth rates | Effects on second-birth rates | Effects on third-birth  rates |
| --- | --- | --- | --- |
| Depression | -0.0042*** (0.0001) | -0.0117*** (0.0005) | -0.0010*** (0.0003) |
| Anxiety | -0.0034*** (0.0002) | -0.0096*** (0.0007) | -0.0012** (0.0004) |
| Schizophrenia | -0.0125*** (0.0003) | -0.0135*** (0.0020) | -0.0045** (0.0017) |
| Bipolar disorder | -0.0059*** (0.0004) | -0.0072*** (0.0014) | -0.0016* (0.0008) |
| Personality disorder | -0.0064*** (0.0003) | -0.0106*** (0.0013) | -0.0017* (0.0008) |
| Control also for partnership status and socioeconomic factors: | | | |
| Depression | -0.0020*** (0.0001) | -0.0065*** (0.0005) | -0.0010*** (0.0003) |
| Anxiety | -0.0009*** (0.0002) | -0.0044*** (0.0007) | -0.0012** (0.0004) |
| Schizophrenia | -0.0045*** (0.0003) | -0.0032 (0.0020) | -0.0049** (0.0017) |
| Bipolar disorder | -0.0027*** (0.0004) | -0.0036* (0.0014) | -0.0019* (0.0008) |
| Personality disorder | -0.0021*** (0.0003) | -0.0037** (0.0013) | -0.0018* (0.0008) |

*Notes*: *p<0.05; ** p<0.01; *** p<0.001

^a^ The models and the samples were as in Appendix Table A6.1, except that they were linear probability models instead of logistic models
